# Impaired NGF/TrkA Signaling Causes Early AD-Linked Presynaptic Dysfunction in Cholinergic Primary Neurons

**DOI:** 10.3389/fncel.2017.00068

**Published:** 2017-03-15

**Authors:** Valentina Latina, Silvia Caioli, Cristina Zona, Maria T. Ciotti, Giuseppina Amadoro, Pietro Calissano

**Affiliations:** ^1^Institute of Translational Pharmacology, National Research Council (CNR)Rome, Italy; ^2^IRCCS Santa Lucia FoundationRome, Italy; ^3^Department of Systems Medicine, University of Rome Tor VergataRome, Italy; ^4^NGF and Molecular Mechanisms of Neurodegenerative Diseases, European Brain Research Institute (EBRI)Rome, Italy

**Keywords:** basal forebrain, septo-hippocampal primary cultures, cholinergic neurons, Alzheimer's Disease (AD), nerve growth factor (NGF), synaptic transmission, electrophysiological recordings, morphological and biochemical analyses

## Abstract

Alterations in NGF/TrkA signaling have been suggested to underlie the selective degeneration of the cholinergic basal forebrain neurons occurring *in vivo* in AD (Counts and Mufson, 2005; Mufson et al., 2008; Niewiadomska et al., 2011) and significant reduction of cognitive decline along with an improvement of cholinergic hypofunction have been found in phase I clinical trial in humans affected from mild AD following therapeutic NGF gene therapy (Tuszynski et al., 2005, 2015). Here, we show that the chronic (10–12 D.I.V.) *in vitro* treatment with NGF (100 ng/ml) under conditions of low supplementation (0.2%) with the culturing serum-substitute B27 selectively enriches the basal forebrain cholinergic neurons (+36.36%) at the expense of other non-cholinergic, mainly GABAergic (−38.45%) and glutamatergic (−56.25%), populations. By taking advantage of this newly-developed septo-hippocampal neuronal cultures, our biochemical and electrophysiological investigations demonstrate that the early failure in excitatory neurotransmission following NGF withdrawal is paralleled by concomitant and progressive loss in selected presynaptic and vesicles trafficking proteins including synapsin I, SNAP-25 and α-synuclein. This rapid presynaptic dysfunction: (i) precedes the commitment to cell death and is reversible in a time-dependent manner, being suppressed by *de novo* external administration of NGF within 6 hr from its initial withdrawal; (ii) is specific because it is not accompanied by contextual changes in expression levels of non-synaptic proteins from other subcellular compartments; (ii) is not secondary to axonal degeneration because it is insensible to pharmacological treatment with known microtubule-stabilizing drug such paclitaxel; (iv) involves TrkA-dependent mechanisms because the effects of NGF reapplication are blocked by acute exposure to specific and cell-permeable inhibitor of its high-affinity receptor. Taken together, this study may have important clinical implications in the field of AD neurodegeneration because it: (i) provides new insights on the earliest molecular mechanisms underlying the loss of synaptic/trafficking proteins and, then, of synapes integrity which occurs in vulnerable basal forebrain population at preclinical stages of neuropathology; (ii) offers prime presynaptic-based molecular target to extend the therapeutic time-window of NGF action in the strategy of improving its neuroprotective *in vivo* intervention in affected patients.

## Introduction

Synaptic dysfunction is a prodromal event in Alzheimer's disease (AD) pathogenesis occurring before neuronal death (Selkoe, 2002) and is more strongly correlated with progressive cognitive impairment than either plaque or tangle pathology (DeKosky and Scheff, 1990; DeKosky et al., 1996; Sze et al., 2000; Scheff et al., 2006, 2007). The basal forebrain system, including the septal region, plays an important role in memory/learning regulating the excitability of the hippocampus and neocortex and is preferentially affected in the initial stages of AD neuropathology (Mesulam, 2004; Schmitz et al., 2016). Cholinergic neurons, which strictly depend on their survival, neuritic outgrowth and phenotype differentiation from target-derived nerve growth factor (NGF) (Hartikka and Hefti, 1988a,b), are progressively reduced in affected patients in correlation with cognitive impairment (Davies and Maloney, 1976; Whitehouse et al., 1982; Wilcock et al., 1982; Lehéricy et al., 1993), supporting the hypothesis that their early-stage disturbance and, in turn, the associated decline in cholinergic innervation of cortical areas are seminal events for cognitive dysfunction occurring in AD. In fact there is a phenotypic downregulation but not a frank loss of cholinergic basal forebrain population in early AD (Grothe et al., 2012, 2013), suggesting that these neurons remain still alive although atrophic up to later stages of the disease progression and, thus, potentially amenable to pharmacotherapeutic interventions aimed to prevent or delay the cognitive/behavioral impairment characterizing the symptomology of this disorder (Mesulam, 2004; Mufson et al., 2008). Consistently, an impaired signaling of NGF is causally linked to selective and extensive loss of central cholinergic functions (Counts and Mufson, 2005; Schliebs and Arendt, 2006; Williams et al., 2006) and NGF replacement therapy has recently emerged as a new promising safety and efficacy disease-modifying treatment for cognitive recovery in patients with mild AD (Tuszynski et al., 2005, 2015; Mufson et al., 2008). In line with the modulatory role of NGF on amyloid precursor protein (APP) secretory pathway(s) in NGF-responsive cells (Rossner et al., 1998; Isacson et al., 2002; Matrone et al., 2008a,b; Calissano et al., 2010a,b), the ectopic administration of NGF stimulates the binding of its high-affinity receptor TrK kinase A (TrkA) to Amyloid Precursor Protein (APP) in *in vitro* cholinergic septal neurons favoring its subcellular localization in Golgi compartment—via downregulation in phosphorylation at the threonine 668 (T668)—which, in turn, reduces susceptibility to BACE cleavage and promotes the anti-amyloidogenic processing (Triaca et al., 2016). NGF supply via nasal route has been proved to *in vivo* modulate the secretase levels and, in turn, reduce the amyloid burden in APP/PS1 transgenic mice (Yang et al., 2014) and transgenic mice lacking the APP-TrkA interaction display forebrain damage and cognitive deficits (Matrone et al., 2012). Cholinergic neurons located in the nucleus basalis of Meynert of affected subjects exhibit a great sensibility to undergo neurofibrillary degeneration at early stages of AD neuropathology (Sassin et al., 2000; Mesulam et al., 2004), suggesting that NGF is also able to influence the tau metabolism, in addition to its effects on basal forebrain cholinergic function(s) and on APP processing (Schliebs and Arendt, 2006). Finally, phenotypic knockout of NGF via its antibody-mediated neutralization in adult transgenic AD11 mice causes age-dependent neurodegenerative changes which are reminiscent of human AD pathology characterized by severe deficits in basal forebrain cholinergic neurons, classic histopathological hallmarks including amyloid plaques and tau neurofibrillary tangles in cortical and hippocampal neurons, behavioral deficits (Capsoni et al., 2000). Therefore, in view of the physiopathological relevance of NGF/TrkA signaling dysfunction in triggering the initial AD-type lesions of vulnerable cholinergic basal forebrain population which critically contribute to memory/learning impairment of hippocampal and cortical areas (Mesulam, 2004), the understanding of the earliest molecular events following neurotrophin starvation in cholinergic septo-hippocampal system will support the development of novel disease modifying drugs aimed to slow down the conversion from asymptomatic Mild Cognitive Impairment (MCI) MCI to clinical full-blown dementia (Mufson et al., 2012).

Although a crucial involvement of early alterations in the NGF/TrkA system in driving neurodegeneration of basalforebrain at the onset of AD progression has been largely accepted, studies carried out on primary septohippocampal cultures have turned out to be technically challenging mainly due to the scarse yield of the cholinergic and TrkA-positive neuronal population transplanted *in vitro* with consequent difficulties in assessing the specificity, the precise timing and, if possible, the reversibility of any biochemical events triggered by NGF withdrawal. By biochemical, morphological and electrophysiological approaches, here we show that the selective reduction of B27(0.2%), the most widely-used serum-free supplement in culture media, combined with chronic somministration of NGF (100 ng/ml), added immediately after plating and for 10–12 days *in vitro* (D.I.V.), significantly increases the number of cholinergic neurons in septal primary cultures (+36.36%) at the expense of non-cholinergic, mainly glutamatergic (−56.25%) and GABAergic (−38.45%), populations. The frequency of spontaneous excitatory miniture post-synaptic currents (mEPSCs) is significantly stimulated upon exposure to ectopic NGF in septohippocampal cultures under conditions of low B27(0.2%) media, confirming that a large amount of cholinergic and NGF-responsive neurons are actually enriched following this experimental procedure. Importantly, by taking advantage of this newly-developed neuronal paradigm, we uncover that the withdrawal of NGF induces a progressive deficit in the presynaptic excitatory neurotransmission which occurs in concomitance with a pronounced and time-dependent reduction in several distinct pre-synaptic markers, such as synapsin I, SNAP-25 and α-synuclein, and in absence of any sign of neuronal death. This rapid presynaptic dysfunction: (i) is reversible in a time-dependent manner, being suppressed by *de novo* external administration of NGF within 6 h from its initial withdrawal; (ii) is specific because it is not accompanied by contextual changes in expression levels of non-synaptic proteins from other subcellular compartments; (ii) is not secondary to axonal degeneration because it precedes the post-translational modifications of tubulin subunits which critically control the cytoskeleton dynamics and is insensible to pharmacological treatment with known microtubule-stabilizing drug such paclitaxel; (iv) involves TrkA-dependent mechanisms because the effects of NGF reapplication are blocked by acute exposure to specific and cell-permeable inhibitor of its high-affinity receptor.

Taken together, these findings suggest that this novel protocol based on defined medium of cell culture provides—with good approximation to the *in vivo* conditions—a significant enrichment/selection in mature and NGF-responsive cholinergic neurons of septal basal forebrain populations, representing thus a unique and relevant *in vitro* AD-relevant model to study the alteration in NGF/TrkA signaling occurring in the vulnerable affected population at pre-symptomatic prodromal stages of disorder. In addition, and more importantly, our results reveal that NGF deprivation acts via TrkA-activation directly and locally on neurosecretory function of cholinergic presynaptic terminals by controlling the steady state levels of synapsin I, SNAP25 and α-synuclein, helping to devise rational therapies that early targets the vulnerable basalforebrain projection system in order to delay and/or reduce the onset of MCI and, ultimately, of clinical AD.

## Materials and methods

### Chemical compounds

Picrotoxin and strychnine were purchased from Sigma (St. Louis, MO) and were dissolved in EtOH and water, respectively. Tetrodotoxin (TTX) was from Alomone Labs (Jerusalem, Israel). DHβE, CNQX and D-AP5 were from Tocris Bioscience (Bristol, UK) and were dissolved in water. NGF was purified from submaxillary glands (Bocchini and Angeletti, 1969). NGF from Xiamen Bioway (Biotech Co., Ltd., Xiamen, Fujian, China) was also used in the study. Recombinant monoclonal antibody to Nerve Growth Factor αD11 (Cattaneo et al., 1988) was generously given from A.Catteneo. TrkA inhibitor GW441756 was from Tocris (Bristol, UK).

The drugs were diluted to their final concentration with the extracellular solution and, unless otherwise stated, the compounds to be tested were added to the perfusion solution. In a separate series of experiments we ensured that final concentration of EtOH had no effect on evoked responses. Solutions containing DHβE, CNQX, D-AP5, and NGF were applied using a multibarrel pipette gravity perfusion system controlled by electronic valves positioned near the soma of the recorded neuron using a fast perfusion system (SF-77B Warner Ins., Hamden, CT, USA; Hatton et al., 2003).

The following antibodies were used: TrkA antibody (763) rabbit sc-118 Santa Cruz; Trk B antibody (794) rabbit sc-12 Santa Cruz; p75 rabbit N3908 Sigma-Aldrich; mAChR M1 antibody (H-120) rabbit sc-9106 Santa Cruz; choactase antibody (H-95) rabbit sc-20672 Santa Cruz; anti-Choline Acetyltransferase antibody goat AB144P Millipore; NMDAζ1 antibody (C-20) goat sc-1467 Santa Cruz; PSD95 antibody (clone 7E3-1B8) mouse MAB1598 Millipore; PSD95 antibody (clone 6G6-1C9) mouse ADI-VAM-PS002-E EnzoLife Science; vGLUT1 antibody rabbit 135,302 Synaptic System; anti-vGAT antibody rabbit 131,003 Synaptic System; mGluR2 antibody rabbit PPS050 RD System; Anti-NeuN antibody (clone A60) mouse MAB377 Millipore; Tau antibody (H-150) rabbit sc-5587 Santa Cruz; Anti-MAP-2 antibody (clone AP20) mouse MAB3418 Millipore; Anti-Synapsin I antibody rabbit AB1543P Millipore; Anti-SNAP-25 antibody (clone SMI 81) mouse 836301 BioLegend; anti-α-synuclein antibody (clone 42) mouse 610786 BD Transduction Laboratories; Dynamin antibody mouse 610246 BD Transduction Laboraories; synaptophysin antibody (D-4) mouse sc-17750 Santa Cruz; anti-syntaxin 1mouse S1172 Sigma-Aldrich; Calnexin antibody (C-20) goat sc-6465 Santa Cruz; VDAC1 antibody (B-6) mouse sc-390996 Santa Cruz; Tom20 antibody (FL-145) rabbit sc-11415 Santa Cruz; acetylated α Tubulin (6-11B-1) mouse sc-23950 Santa Cruz; rat anti tubulin alpha mouse MCA77G Bio-Rad; Anti-caspase 3 cleaved (active) form antibody rabbit AB3623 Millipore; AβPP antibody aa 66-81 (22C11) mouse MAB348 Millipore; AβPP antibody (4G8) aa 18-22 of the Aβ peptide mouse Covance SIG39220; GAPDH antibody (clone GAPDH-71.1) mouse G8795 Sigma-Aldrich; β-actin mouse S3062 Sigma-Aldrich; anti-mouse IgG (whole molecule)-Peroxidase antibody A4416 Sigma-Aldrich; anti-rabbit IgG (whole molecule)-Peroxidase antibody A9169 Sigma-Aldrich; donkey anti-goat IgG-HRP antibody sc2056 Santa Curz.

### Animals

Pregnant Wistar rats (Charles River Laboratories) at gestational age 17-18 (E17-18) were used for all the experiments of this study. Experimental protocols were carried out in accordance with the European Communities Council Directive of 22 September 2010 (2010/63/UE) regarding the care and use of animals for experimental procedures and with the Italian legislation on animal experimentation (Decreto L.vo 116/92).

### Septal neurons primary cultures

Septal neurons were prepared from embryonic day 17/18 (E17/18), as previously described (Hartikka and Hefti, 1988a,b) with some modifications. Briefly, embryos were surgically removed and septo-hippocampal areas were dissected from the cerebral tissue in ice-cold Hanks' balanced salt solution (HBSS, Gibco, Life Technologies), freed of meninges, digested with 0.25% trypsin for 15 min at 37°C, dissociated by trituration and seeded as follows: 2 × 10^6^ cells on poly-l-lysine (SIGMA)-coated plates (BD Falcon, Durham, NC, USA; 353001) for biochemistry analyses and 10 × 10^4^ cells on glass coverslips in 24-well plates (BD Falcon; 351147) for immunofluorescence analyses. The dissociated cells were plated serum-free Neurobasal medium (Gibco, Life Technologies) supplemented with B27 (Invitrogen Inc., Carlsbad, CA, USA) at final 2 or 0.2% concentration in the presence of NGF (100 ng/ml) for 10–12 days. One day after plating, cytosine arabinoside (5 μM) was added to inhibit glial proliferation. Cultures were kept at 37°C in a humidified incubator in a 5% CO_2_ atmosphere without further medium changes until used for experiments.

### Assessment of neuronal viability

Cell viability was quantified by assessing cytomorphological and biochemical alterations, including the count of the number of apoptotic nuclei (Loo and Rillema, 1998), the mitochondrial MTT tetrazolium salt assay (Manthorpe et al., 1986), the Western blotting detection of active cleaved caspase-3 (Asp175) (Srinivasan et al., 1998).

### Immunofluorescence

Following treatment, septohippocampal cultures were washed twice with PBS and fixed in 4% (w/v) paraformaldehyde for 15 min at room temperature. Cells were permeabilized with 0.1% (v/v) Triton X-100/PBS pH 7.4 for 4 min at room temperature. Coverslips were saturated with 2% BSA and 10% normal goat serum (NGS) for 3 h followed by incubation overnight at 4°C in a humidified chamber with primary antibodies. Unbound antibody was removed by three washes and bound antibody was detected by incubation with donkey anti-mouse-488 and donkey-anti-rabbit-555 IgG from Jackson ImmunoResearch (1:300), or donkey anti-goat-488 and donkey-anti-rabbit-594 from Life Technologies (1:500) at room temperature for 30 min. Nuclei were stained with nuclear marker 4,6-diamidino-2-phenylindole dihydrochloride (DAPI; Sigma, St. Louis, MO, USA) 1:1,000 in PBS for 5 min and samples were mounted on glass slides, and cover slipped with antifade medium. Controls were performed by omitting either the primary or the secondary antibody. Images are representative of at least three independent experiments and were acquired with an Eclipse 80i Nikon Fluorescence Microscope (Nikon Instruments, Amsterdam, Netherlands).

### Protein cellular lysates preparation

Total proteins were extracted by scraping the cells in ice-cold RIPA buffer (50 mM Tris-HCl pH 8, 150 mM NaCl, 1% NP40, 0,1% SDS, 5% sodium deoxicholate plus proteases inhibitor cocktail (Sigma Aldrich, P8340) and phosphatase inhibitor cocktail (Sigma Aldrich, P5726/P2850) and centrifuged at 4°C for 20 min at 13,000 rpm. The supernatant was then collected and the amount of total protein was determined by Bradford assay (Protein Assay Dye Reagent Concentrate, BioRad, Hercules, CA, USA).

### SDS-PAGE, western blot analysis and densitometry

Equal amounts of protein were separated by SDS-PAGE in 4–12% Bis-Tris gels (Invitrogen), and transferred to nitrocellulose membranes (0.45 μm, GE healthcare, Little Chalfont, UK). The filters were blocked in TBS-T containing 5% non-fat dried milk for 1 h at room temperature or overnight at 4°C. Proteins were visualized using appropriate primary antibodies. All primary antibodies were diluted in TBS-T and incubated with the nitrocellulose blot overnight at 4°C. Incubation with secondary peroxidase coupled anti-mouse, anti-rabbit or anti-goat antibodies was performed by using the ECL system (Thermo Scientific SuperSignal West Pico, 34080; Amersham ECL Prime, RPN2232). Protein loading was monitored by normalization to β-actin. Blots were scanned and quantitative densitometric analysis was performed by using ImageJ (http://imagej.nih.gov/ij/).

### Electrophysiological recordings

Whole cell patch-clamp recordings were performed from 10 to 12 day-old *in vitro* (D.I.V.) primary septum neurons to study the excitatory neurotransmission under different culture conditions (Caioli et al., 2011). Particularly, the neurons were voltage clamped at −60 mV. The recording pipettes were pulled from borosilicate glass with an outer diameter of 1.2 mm and had open tip resistances of 3–5 MΩ. The internal solution for filling pipettes consisted of (in mM): 140 CsCl, 1 EGTA, 10 HEPES, 6 D-glucose (pH 7.4 with CsOH). The standard extracellular solution consisted of (in mM): 130 NaCl, 3 KCl, 2 MgCl_2_, 1.5 CaCl_2_, 10 HEPES, 6 D-glucose, and 10 tetraethyl-ammonium (TEA) Cl (pH 7.4 with NaOH).

To isolate the miniature Excitatory Post Synaptic Currents (mEPSCs), 0.5 μM tetrodotoxin (TTX), 5 μM strychnine, 100 μM picrotoxin were added in the bath solution, in order to block voltage-dependent Na^+^ currents, glycine and GABAA receptors, respectively. Recordings were carried out for 5 min from each neuron and the last 2 min of each recording were analyzed.

In order to evaluate the contribution of the cholinergic and glutamatergic activity in the excitatory neurotransmission, the competitive nicotinic acetylcholine receptor antagonist dihydro-β-erythroidine hydrobromide (DHβE; 50 μM) was applied to some neurons. In particular, following 3 min of total mEPSC recording (control condition), DHβE was applied to each neuron for 3 min and the last 2 min of each recording were analyzed. The remaining mEPSCs were abolished completely by 5 μM 6-cyano-7-nitroquinoxaline-2,3-dione (CNQX) and 10 μM D-2-amino-5-phosphonovalerate (D-AP5).

In some experimental conditions, the short term effect of NGF on mEPSCs was tested by applying NGF (100 ng/ml) directly on the recorded neurons. Following 3 min of total mEPSC recording (control condition), NGF was acutely applied for 3 min and the last 2 min of each recording were analyzed. In some experiments, TrkA inhibitor (15 μM) was added to the culture medium for 20 min, prior to start the electrophysiological experiments, and then was added to the extracellular solution, in order to selectively block the NGF receptor.

Experiments were performed at room temperature (22–24°C). After the formation of a high-resistance seal (>1 GΩ), the capacitance and the resistance of the electrodes were compensated electronically. Recordings were made using a MultiClamp 700B amplifier (Axon CNS, Molecular Device). pCLAMP 9.2 software was utilized for the data acquisition system (Axon Instruments). The whole cell capacitance was assessed online using the Membrane test function of pClamp9.2. Current signals were sampled at 100 kHz and filtered at 3 kHz.

### Data analysis

Data presented are representative of at least three independent experiments and were expressed as mean ± SEM. Statistical analysis of all the experiments in this study were performed by GraphPad Prism (GraphPadSoftware), SPSS 17.0.0 for Windows (SPSS Inc., Chicago, IL, USA), Origin 7.0 (Microcal Software, Northampton, MA, USA) using appropriate test, i.e., paired or unpaired Student's *t*-test and One-Way ANOVA followed by Bonferroni *post hoc* correction. Results were considered significant when *p* < 0.05. To analyze miniature post synaptic currents (mEPSCs), the 6.0.7 version of Mini Analysis Program (Synaptosoft Inc., Decatur, GA, USA) was used. mEPSCs were manually detected using a 8 pA threshold crossing algorithm. Frequency, event amplitude, kinetic characteristics (rise and decay time), and event area were compared between the different experimental conditions.

## Results

### Chronic NGF treatment of septo-hippocampal cultures in 0.2% B27 media selectively enriches for cholinergic and TrkA-expressing target neuronal population

**Figure 1:**
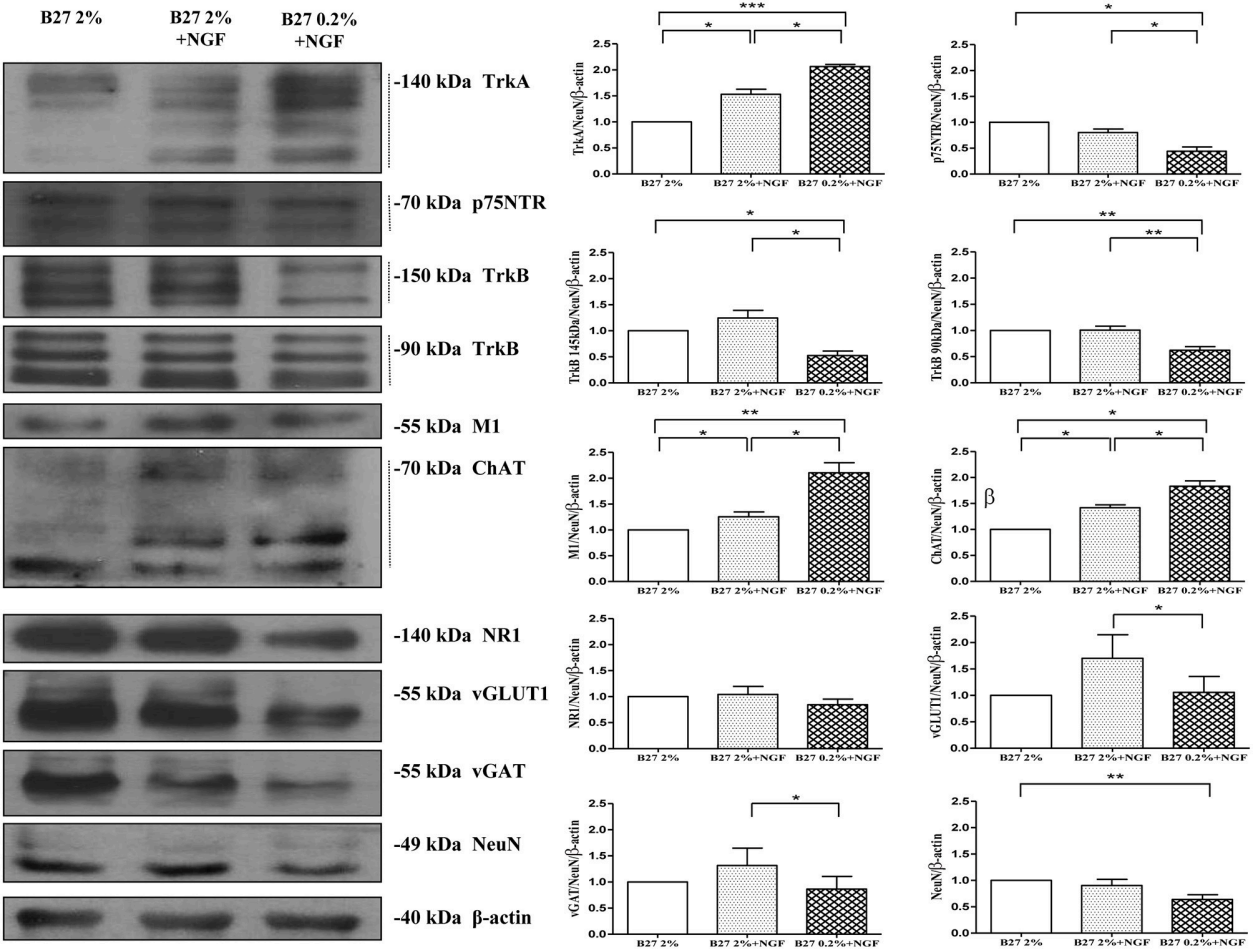
**Biochemical characterization in the expression profile of glutamatergic, GABAergic cholinergic phenotype-specific markers in septohippocampal cultures grown in the chronic presence of NGF (100 ng/ml) under different conditions of B27 supplementation**. Western blotting analysis (*n* = 9) was carried out on equal amounts of total protein extract (20–80 μg) from septal primary neurons cultured for 10–12 D.I.V. in defined medium supplemented with 2% B27 or 0.2% B27, in the presence or absence of exogenously-added NGF (100 ng/ml) Immunoblots were probed with antibodies against specific markers of cholinergic (TrkA high-affinity NGF receptor, p75NTR low-affinity NGF receptor, TrkB BDNF receptor, muscarinic acetylcholine receptor M1, choline acetyltransferase ChAT) glutamatergic (N-methyl-D-aspartate glutamate receptor 1 NR1, vesicular glutamate transporter 1 vGLUT-1) and GABAergic (vesicular γ-AminoButyric Acid transporter vGAT) phenotypes to compare the extent of each neuronal population among three different culture conditions. Cropped representative WB are shown. Molecular weights are indicated on the right of the blots and expressed in kDa. Densitometric quantification of immunoreactivity levels were calculated by normalizing the values on the intensity ratio of β-actin, as loading control for each sample/lane, and NeuN, as neuron-specific marker to minimize the glial cells component (less than 5%). Dashed line indicates the bands used for the quantification. Values are mean ± standard error of the mean (SEM) of at least nine independent experiments and are expressed with respect to B27 2% control neurons. Statistically significant differences were calculated by unpaired-two tailed *t*-Student's test (^*^p < 0.05, ^**^p < 0.01 and ^***^p < 0.0001). Note that the defined medium supplemented with 0.2% B27 in the chronic (10–12 D.I.V.) presence of NGF (100 ng/ml) promotes the selective enrichment in cholinergic NGF-responsive neuronal population in septo-hippocampal primary cultures.

The medial septal region (medial septum and diagonal band of Broca, MS/DB), which controls hippocampal excitability and synaptic plasticity associated to learning and memory (Olton et al., 1978; Poucet and Herrmann, 1990; Dutar et al., 1995; Niewiadomska et al., 2009), includes different neuronal subpopulations with heterogeneous morphology, biochemical, electrophysiological properties and connectivity. In fact, a detailed characterization of the *in vivo* septo-hippocampal projections has recently given strong evidence of three distinct neurotransmitter pathways including GABAergic (27.8%), cholinergic (47%) and glutamatergic (24.5%) neurons (Colom et al., 2005, 2013; Wu and Yeh, 2005; Huh et al., 2008). However, as cholinergic neurons represent only a part of distinct neuronal subpopulations located in the septo-hippocampal circuit (Colom et al., 2005, 2013; Huh et al., 2008; Teles-Grilo Ruivo and Mellor, 2013), basic and pharmacological AD research will benefit from their selective and robust enrichment in order to be studied in the absence of any other neuronal and non-neuronal contaminants. On the other hand, the setting-up of an experimental method which provides with a high-degree of homogeneity good yields of septal primary cultures fully-mature at both morphological and functional levels is particularly troublesome to technically overcome because post-mitotic neurons do not proliferate. Therefore, with the intent of developing a novel *in vitro* tool which both ricapitulated the *in vivo* conditions and enabled to assess without any confusing off-target effects the direct action of NGF starvation on a more consistent and genuine cholinergic population, primary neurons from dissociated septal sections of fetal (E17) rat containing MS/DB basal forebrain area were chronically maintained in culture media with different concentrations of B27 supplementation (2%, 0.2%) in the absence or in presence of exogenously-added NGF (100 ng/ml). Importantly, the *in vitro* experimental paradigm we used in the present work has been designed to ensure that the specific action of extracellularly-added NGF was restricted to its physiological target population. In fact, since cholinergic neurons of the basal forebrain are the only ones in the brain to constitutively express both the requisite Tropomyosin kinase A (TrkA) and p75 neurotrophin (p75NTR) receptors (Sobreviela et al., 1994), they are uniquely sensitive to administration of ectopic NGF. After 10–12 D.I.V., Western blotting analysis on total protein extracts were performed by probing with antibodies against specific markers of cholinergic (TrkA high-affinity NGF receptor, p75NTR low-affinity NGF receptor, TrkB BDNF receptor, muscarinic acetylcholine receptor M1, choline acetyltransferase ChAT) glutamatergic (N-methyl-D-aspartate glutamate receptor 1 NR1, vesicular glutamate transporter 1 vGLUT-1) and GABAergic (vesicular γ-AminoButyric transporter vGAT) phenotypes to compare the extent of each neuronal-type population among three different culture conditions (2% B27, 2% B27+NGF, 0.2% B27+NGF). As shown in Figure 1 by biochemical characterization and relative densitometric quantification, septal primary neurons grown for 10–12 D.I.V in classic media supplemented with 2% B27 and in the absence of NGF (2% B27experimental group) showed a sizeable but detectable expression of three different well-confirmed cholinergic-specific markers such as M1, ChAT and TrkA (Dawbarn et al., 1988; Holtzman et al., 1992; Sobreviela et al., 1994), in line with previous findings reporting that differentiation and initial survival of central NGF-responsive neurons in the early postnatal period can occur even in the absence of NGF (Crowley et al., 1994; Ruberti et al., 2000) and that glial astrocytes are able to synthesize NGF and secrete it in culture (Lindsay, 1979; Norrgren et al., 1980; Furukawa et al., 1986). The addition of exogenous NGF (100 ng/ml) for the same period of time (10–12 D.I.V.) to B27-rich cultures media (2% B27+NGF experimental group) increased the specific expression profile of cholinergic phenotype of septal neurons, consistent with its renowned and potent action in positively stimulating the differentiation of the target responsive population in high-density cultures of basal forebrain (Hartikka and Hefti, 1988a,b; Kew and Sofroniew, 1995). Interestingly, we found out that a more robust upregulation in M1, ChAT, and TrkA protein levels was detected when neuronal population was shifted to media supplemented with lower B27 concentration and in the presence of extracellular NGF (100 ng/ml; 0.2% B27+NGF experimental group) in comparison with sister 2% B27 control cultures. Conversely, the immunoreactivity of other not-cholinergic markers -such as NR1, vGLUT-1 and vGAT- was contextually reduced in 0.2% B27+NGF experimental group, suggesting that this newly-developed culture procedure relying on lower B27 supplementation was actually able to enrich for a more copious NGF-responsive cholinergic septo-hippocampal population. Furthermore, the extent of TrkB expression—another neurotrophic tyrosine kinase receptor which also supports the survival and phenotypic differentiation of basal forebrain cholinergic neurons upon interaction with BDNF in a ligand-specific manner (Alderson et al., 1990)—was not positively affected following this culture protocol consistent with previous findings showing that rat septal cultures that respond to BDNF or NGF are closely overlapping but not the similar populations (Alderson et al., 1990; Colom et al., 2005). Likewise, no significant change was contextually detected in the intensity signal of the low-affinity NGF receptor p75NTR, in line with the observation that 95% of septal cholinergic neurons express TrkA (Sobreviela et al., 1994) and that p75NTR does not play a major *in vivo* role in the maintenance of the number or morphology of medial septum cholinergic neuron (Ward et al., 2000). It's worth pointing out that, although NGF is capable of stimulating both the mRNA and the protein expression of its own receptor(s) (Montero and Hefti, 1988; Higgins et al., 1989; Holtzman et al., 1992) and the enzymes associated with regulation of acetylcholine amount (ChAT and AChE) (Honegger and Lenoir, 1982; Gnahn et al., 1983; Mobley et al., 1986; Alderson et al., 1989; Li et al., 1995), only the long-term somministration of exogenous NGF for 10–12 D.I.V. combined with a lower concentration of B27 supplementation turned out to be effective in significantly improving the cell-type density, likely in a synergistic way. Taken together, these findings suggest that the selective enrichment in cholinergic population under adverse *in vitro* conditions of lower doses of nutrients is to be ascribed to the *de novo* induction in expression of NGF-stimulated specific markers in close association with its survival action on TrkA-expressing responsive neurons, as supported by the significant diminution in viability (ranging from 30 to 60%, as assessed by MTT assay and Hoechst nuclei staining respectively) we detected comparing the two experimental groups kept continously in presence of NGF for 10–12 D.I.V. but in media supplemented with different concentrations of B27 (Supplentary Figure 1). Furthermore, an unknown soluble nutritional B27 component -perhaps insulin- may also act in addition to NGF as general growth factor being able to maintain the *in vitro* survival of all three neuronal populations but not the phenotype of cholinergic neurons, in line with previous observations (Svendsen et al., 1994; Kew and Sofroniew, 1995).

**Figure 2:**
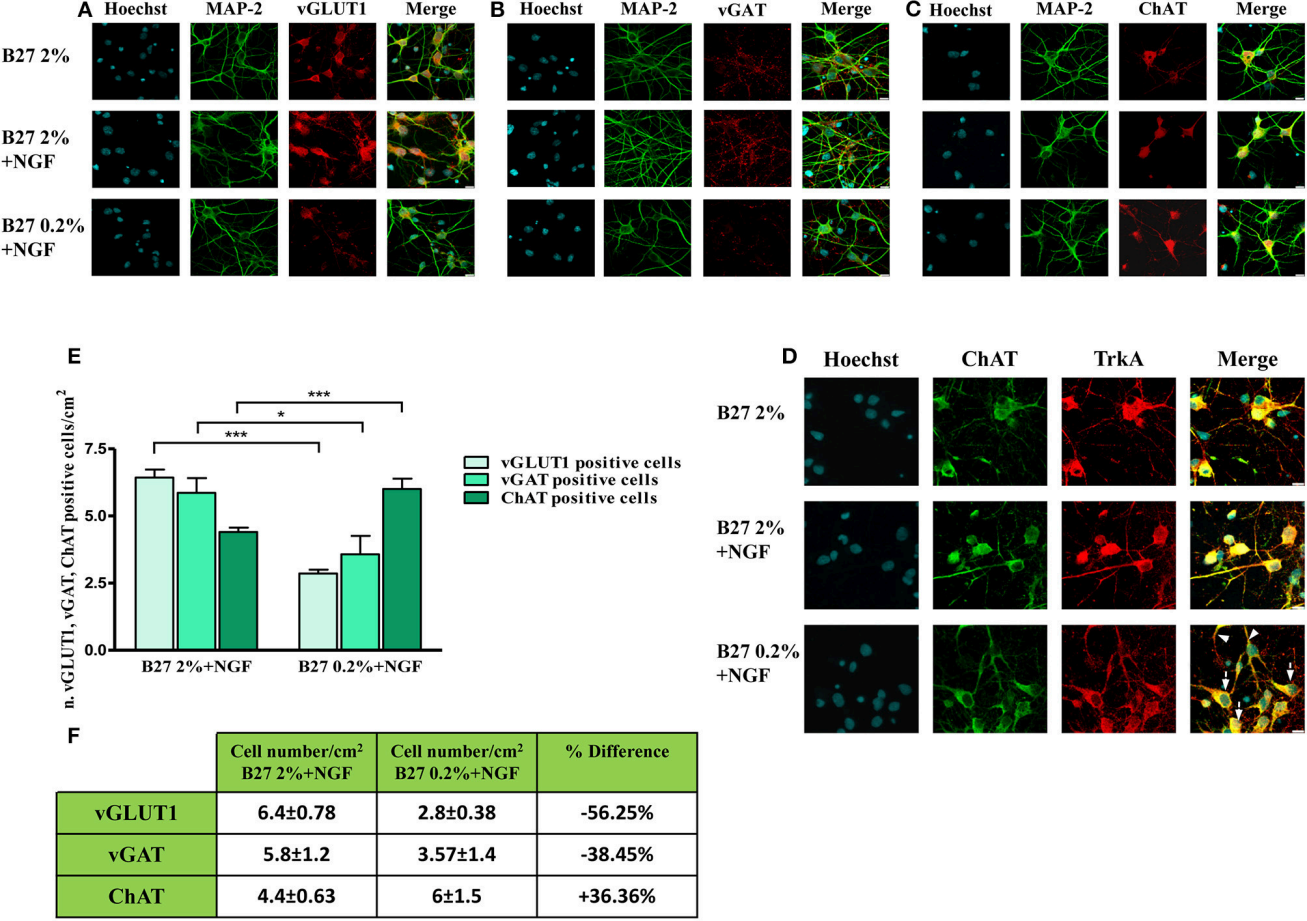
**Selective reduction in the size of non-cholinergic, mainly glutamatergic and GABAergic, neuronal populations are detected in septal neurons exposed for 10–12 days *in vitro* to 100 ng/ml NGF in B27 0.2% culturing medium. (A–D)** Confocal microscopy analysis of double immunofluorescence carried out on septohippocampal primary neurons cultured for 10–12 D.I.V. in defined medium supplemented with 2% B27 or 0.2% B27, in the presence or absence of exogenously-added NGF (100 ng/ml). Merge images show the overlay of the two fluorescence channels. Staining for dendritic MAP-2 enabled to visualize the integrity of neuritic network whereas labeling for phenotype-selective marker was used to identify the extent of each specific neuronal population in septal primary cultures. **(A)** Dendritic MAP-2 (green channel) and glutamatergic-specific marker vGLUT-1 (red channel); **(B)** Dendritic MAP-2 (green channel) and GABAergic-specific marker vGAT (red channel); **(C)** Dendritic MAP-2 (green channel) and cholinergic-specific marker ChAT (red channel); **(D)** Two cholinergic-specific markers, ChAT (green channel) and TrkA (red channel). Nuclei (blue) were stained with Hoechst 33258 (0.5 mg/ml). Note the loss in vGLUT1- and vGAT-positive neurons under the B27 0.2% +NGF experimental conditions of culturing media. Images are representative of at least three independent experiments. Scale bar: 10μM. **(E,F)** Graph represents the averaged number ± SEM. (*n* = 5–10) of of vGLUT1- vGAT- and ChAT-positive neurons per 100 X image-field in septohippocampal cultures grown in the chronic presence of NGF(100 ng/ml) under different conditions of B27 supplementation. The number of each cell type was counted under conditions of B27 2% nutrients and compared with the number present in sister wells grown B27 0.2%-supplemented media. Statistically significant differences were calculated by unpaired-two tailed *t*-Student's test (^*^p < 0.05 and ^***^p < 0.0001 between corresponding cell numbers at the experimental time-point). Table represents the mean of number distribution (number of cells/cm^2^) and % variation of neurons present in the defined medium at the experimental time-point. Means ± SEM. are given. These results demonstrate a significant increase in cholinergic neurons (+36.36%) at the expense of all other non-cholinergic populations of septum -mainly GABAergic (−38.45%) and glutamatergic (−56.25%) populations.

Next, in order to better visualize the actual size of cholinergic and non-cholinergic -mainly glutamatergic and GABAergic- subpopulations under these three different *in vitro* culture conditions (2% B27, 2% B27+NGF, 0.2% B27+NGF), we carried out a morphological chracterization of septo-hippocampal neurons by double-immunocytochemistry analysis with an antibody against microtubule associated protein 2 MAP2 -a neuron-specific marker which decorates perikarya and dendrites processes- in association with one of the three different phenotype-selective antibodies directed against TrkA, vGLUT1, vGAT respectively. As shown in Figures 2A–C and consistently with Western blotting results, a selective and substantial reduction in the proportion of vGLUT-1 or vGAT-positive neurons was detected when septal cultures were exposed for 10–12 D.I.V. to 100 ng/ml NGF in 0.2% B27 media (−56.25 and −38.45%, respectively). Furthermore, under these growth conditions, cell number quantification (Figures 2E,F) showed a noteworthy increase in the total number of neurons which were double-stained for MAP-2 and ChAT, indicating a marked enrichment (+36.36%) in NGF-sensitive cholinergic population whose percentage has known to be very low in basal forebrain primary cultures when long-term kept in canonical high-concentrated 2% B27 culture media (Hartikka and Hefti, 1988a,b; Alderson et al., 1990; Svendsen et al., 1994). To better characterize the identity of cholinergic population, a double-immunocytochemistry analysis for the two phenotype-specific ChAT and TrkA markers (Dawbarn et al., 1988; Holtzman et al., 1992; Sobreviela et al., 1994) was also performed on septal neurons cultured under these different *in vitro* conditions from plating. As shown in Figure 2D, in 2% B27 culture media both in the absence and presence of exogenous NGF, only few ChAT/TrkA- double positive neurons could be detected *in vitro* ranging from 1 to maximally 5% of all cell population, as previously reported (Hartikka and Hefti, 1988a,b; Svendsen et al., 1994). In contrast, prolonged NGF treatment in media supplemented with low 0.2% B27 nutrients significantly increased the number per dish- and to some degree the staining intensity as well- of individual cholinergic neurons decorated with diffuse ChAT and TrkA immunolabeling which codistributed with similar pattern along cell-body and neuritic processes of both high and low caliber (Figure 2D, arrows and arrowheads respectively). In line with previous findings (Hartikka and Hefti, 1988a,b; Svendsen et al., 1994), we confirmed that *in vitro* septal cholinergic neurons usually displayed rounded cell bodies and were larger than those expressing other neurotransmitter(s) whose shape included oval and polygonal/pyramidal configurations, although the punctated immunoreactivity pattern of vGAT and vGLUT1 antibodies made sometimes difficult the univocal visualization of neuronal soma. Furthermore and consistent with previous reports (Hartikka and Hefti, 1988a,b; Colom et al., 2005), all ChAT-positive neurons were also costained for TrkA and did not immunoreact with vGLUT-1 and vGAT with the exception of a small minority of neurons that were labeled with more than one single antibody, as we also detected in our triple immunolabeling analysis (data not shown). Finally, an evident increase in proportion of dense heterochromatin nuclei was detected in neuronal cultures grown in 0.2% B27+NGF media in keeping with the cell viability assays (Supplementary Figure 1), supporting thus the notion that when septal cultures were maintained for long periods of time (10–12 D.I.V.) under these *in vitro* conditions which favored survival and morphological features of cholinergic phenotype a gradual and selective loss of non-cholinergic neurons at the expense of ChAT and TrkA-positive population occurred.

Taken together, our biochemical and morphological results demonstrate that: (i) treatment with NGF(100 ng/ml) under *in vitro* conditions of low 0.2% B27 nutrients selectively keeps alive forebrain cholinergic neurons at the expense of all other non-cholinergic resident populations—mainly GABAergic (−38.45%) and glutamatergic (−56.25%)—accounting for their progressive significant enrichment (+36.36%) in primary septal cultures after 10–12 D.I.V.; (ii) the combined survival- and growth-enhancing selection sustained by the ectopic addition of NGF in the presence of 0.2% B27 supplementation provides a simple, less expensive and valuable method to obtain a consistent and genuine cholinergic population which can be advantageous for *in vitro* studies aimed to investigate the survival/disease changes of basal forebrain septo-hippocampal projections occurring during the prodromal stages of AD pathology and caused by dysfunction in NGF/TrkA signaling.

### Long-term *In vitro* cholinergic neurons cultured in the presence of NGF under conditions of 0.2% B27 supplementation acquire mature electrophysiological properties that closely resemble those of *In vivo* central nervous system (CNS) neurons

Compelling evidence has proved that *in vivo* intraseptal infusion of NGF influences the cholinergic neuronal morphology, hippocampal plasticity and behavior in the intact rat brain, suggesting that one of the physiological functions of this neurotrophic factor in adult CNS is the dynamic modulation of strength of cholinergic projections to hippocampal/cortical targets and, ultimately, of the neuronal plasticity (Conner et al., 2009). In support of this statement, it has been demonstrated that an increase in NGF availability significantly stimulates the septal cholinergic cell function(s) and facilitates the induction of hippocampal long-term potentiation (LTP) whereas its reduction suppresses cholinergic-type markers (Sofroniew et al., 1990) and impairs retention of spatial memory (Conner et al., 2009). In keeping with its *in vivo* neuromodulatory function, it has also been shown that NGF is able to selectively facilitate the synaptic transmission of cholinergic basal forebrain primary neurons without affecting the other resident noncholinergic populations- mainly glutamatergic and GABAergic- which express different neurotransmitter phenotypes (Wu and Yeh, 2005; Huh et al., 2008). Consistently, chronic exposure of MS-DB primary cultures to NGF leads to an increase in the neurotransmitter release but not in the density of postsynaptic receptors—likely by rapid control of local protein synthesis (Kang and Schuman, 1996; Melemedjian et al., 2010)—indicating that this neurotrophin is able to exert a potent and specific stimulatory presynaptic action on cholinergic nerve terminals (Huh et al., 2008). In view of these observations, the status of excitatory neurotransmission of septo-hippocampal neurons cultured under media conditions of B27 0.2% supplementation in the chronic presence of NGF (100 ng/ml) was investigated in order to check whether the ensuing cholinergic-enriched population was also endowed with appropriate functional properties of NGF responsiveness. To this aim, we performed electrophysiological recordings of spontaneous miniature excitatory post-synaptic currents (mEPSCs), which are largely accepted to rise from random release of presynaptic vesicles at nerve endings and to play important roles in maintaining functional connections of synaptic terminals (Caioli et al., 2011; Saba et al., 2016).

**Figure 3:**
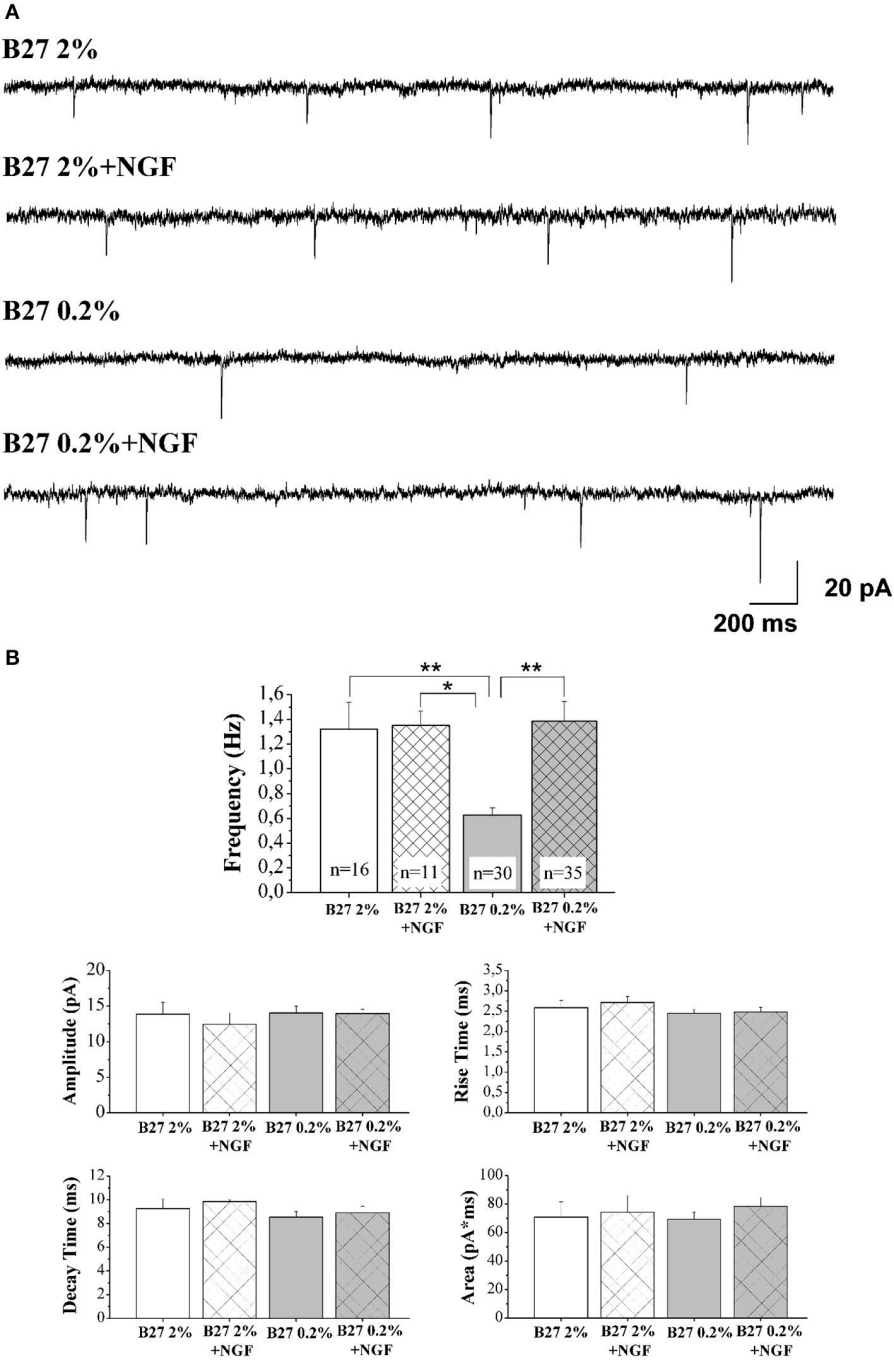
**Septal primary neurons cultured for 10–12 D.I.V. in defined medium supplemented with 0.2% B27 and in the presence of exogenous NGF (100 ng/ml) acquired NGF-dependent neurophysiological properties, just resembling those of *in vivo* mature cholinergic neurons. (A)** Representative traces of mEPSCs recorded from septal neurons grown for 10–12 D.I.V. in B27 2%, B27 2%+NGF, B27 0.2%, and B27 0.2%+NGF culture media. **(B)** Bar plots report the mean ± SEM frequency, amplitude, rise time, decay time and area of the mEPSCs recorded under different experimental conditions. Primary cultures grown in B27 0.2%+NGF medium (B27 0.2%+NGF: 1.38 ± 1.16 Hz) showed averaged mEPSC frequency significantly higher than that from neurons kept in B27 0.2% (B27 0.2%: 0.63 ± 0.06 Hz, *n* = 30) whereas not significant difference was found in comparison to B27 2% and B27 2%+NGF (B27 2%: 1.32 ± 0.21 Hz, *n* = 16; B27 2%+NGF: 1.35 ± 0.11 Hz, *n* = 11). Values are means of at least four independent recordings and statistically significant differences were calculated by One-Way ANOVA followed by Bonferroni's correction. (^**^p < 0.01 vs. B27 2% and B27 0.2%+NGF); (^*^p < 0.05, vs. B27 2%+NGF). No significant differences were detected for the other analyzed parameters.

In Figure 3A are reported representative mEPSCs of septo-hippocampal neurons kept for 10–12 D.I.V. under different culture conditions, including B27 2%, B27 2%+NGF, B27 0.2%, and B27 0.2%+NGF, recorded at the holding potential −60 mV (see methods for details). As shown in Figure 3B, the mean of the mEPSC frequencies was significantly reduced in neurons grown in conditions of low B27 supplementation (B27 0.2%: 0.63 ± 0.06 Hz; *n* = 30) in comparison with sister cultures kept for the same duration with a high dose of B27 nutrients (B27 2%: 1.32 ± 0.21 Hz; *n* = 16; B27 2%+NGF: 1.35 ± 0.11 Hz, *n* = 11 *p* < 0.01, One-Way ANOVA). These results indicate that the extent of excitatory neurotransmission of septal neurons was *per se* significantly affected by the diminution of the B27 concentration regardless of the modulatory presynaptic effect of NGF, likely due to its negative impact on cell survival that we detected to be less than 30% in *in vitro* conditions of B27 0.2% alone (data not shown). Interestingly, exogenously-added NGF (100 ng/ml) was able to strongly enhance the excitatory neurotransmission when septo-hippocampal populations were chronically grown in its presence under B27 0.2% conditions, as evidenced by the fact that the mean of mEPSC frequencies of neurons kept in B27 0.2%+NGF medium (1.38 ± 0.16 HZ; *n* = 35) was significantly higher to that of corresponding sister cultures maintained under conditions of B27 0.2% alone (*p* < 0.05, One-Way ANOVA). Furthermore, no significant difference was detected in the average of mEPSCs frequencies between septal cultures kept in B27 0.2%+NGF medium and those grown under conditions of high B27 nutrients (2%), both in the presence and absence of NGF (*p* > 0.05, One-Way ANOVA).

Finally, in line with previous data reporting a specific effect of this neurotrophin on *in vitro* cholinergic neurons at the presynaptic level (Wu and Yeh, 2005; Huh et al., 2008), the mean amplitude as well as the mean area and the kinetic parameters of the mEPSCs were not modified, both by B27 medium concentrations and by treatment with exogenous NGF (*p* > 0.05, One-Way ANOVA). The findings that the averaged frequency of miniture events detected in neuronal cultures grown under conditions of 0.2% B27 + NGF, despite a consistent reduction in their cell viability (30–50%) which *per se* could negatively impact on the extent of recorded afferent inputs (Supplementary Figure 1), was comparable to that of corresponding ones kept in B27 2% independently of the presence of extracellularly-added NGF, clearly indicated that a larger proportion of cholinergic and, then, uniquely NGF-responsive (Sobreviela et al., 1994) neurons was actually *in vitro* enriched following this culture protocol.

In order to quantify the relative contribution of the cholinergic and glutamatergic neurotransmission in the mEPSCs recorded in 0.2% septum primary neurons, we pharmacologically isolated the two components by means of DHβE (50 μM), a selective competitive nicotinic receptor antagonist, and CNQX (5 μM) and D-AP5 (10 μM), two potent competitive antagonist of AMPA/kainate receptor and NMDA receptor respectively. In detail, for each neuron the total mEPSCs were recorded for 3 min, DHβE was applied for the following 3 min and then the remaining glutamatergic mEPSCs were analyzed for the last 2 min of each recording. Next, the activity was further blocked by CNQX (5 μM) and D-AP5 (10 μM) and the mEPSC frequency post-DHβE perfusion was normalized with that in control condition for each neuron to quantify the glutamatergic component of mEPSCs, while the remaining component was considered as cholinergic activity.

**Figure 4:**
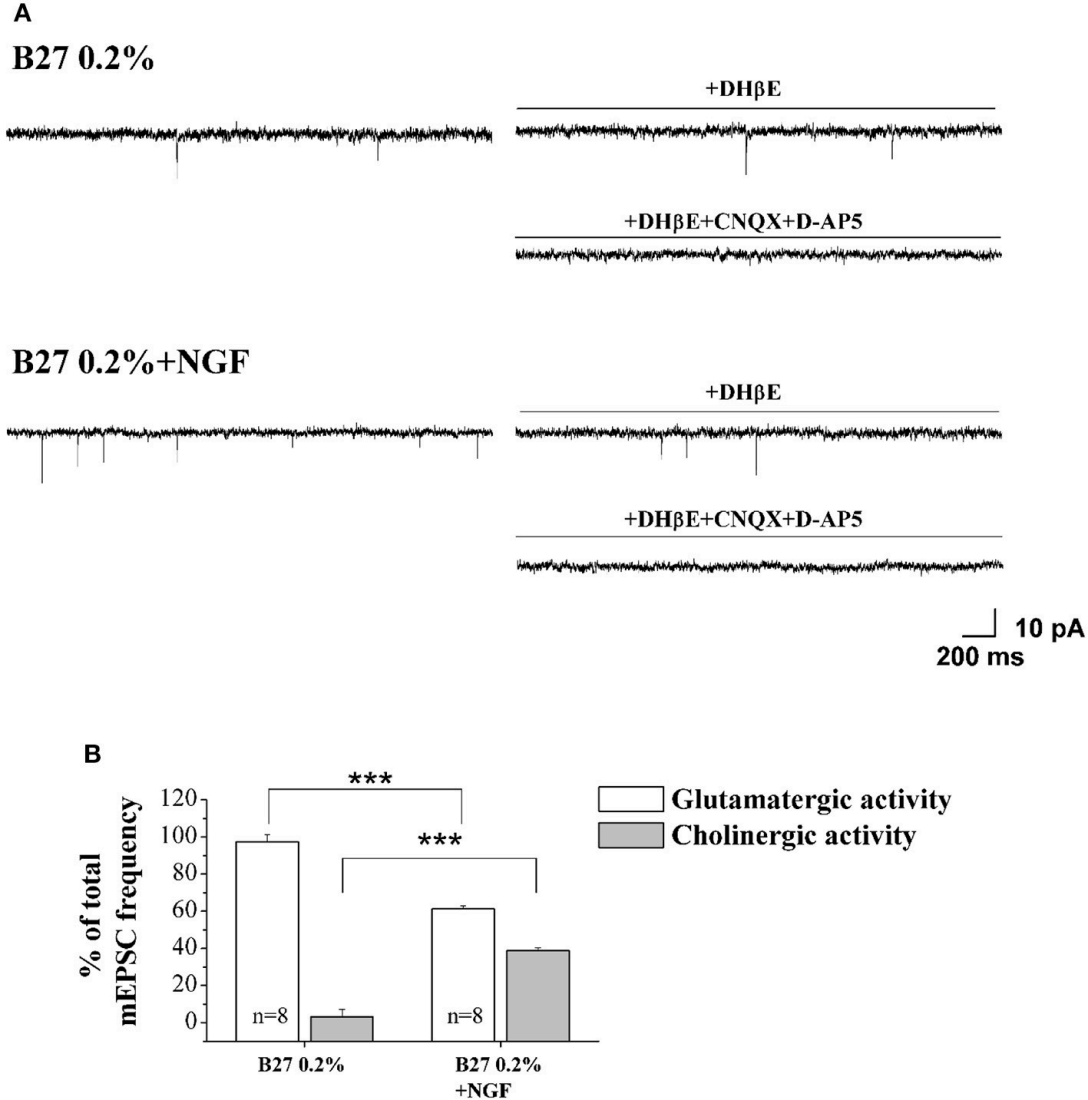
**Cholinergic neurons cultured in 0.2% B27-supplemented media and in the chronic (10–12 D.I.V) presence of exogenously-added NGF (10 ng/ml) expressed mEPSCs with larger frequency than control cholinergic population kept for the same duration under conditions of 2% B27 nutrients (A)** Representative traces of septal primary neurons grown for 10–12 D.I.V. in B27 0.2% and in B27 0.2%+NGF, in basal settings, following DHβE (50 μM; 3 min) and DHβE+CNQX (5 μM)+D-AP5 (10 μM) perfusion (3 min). **(B)** Bar plots report the mean ± SEM percentage of glutamatergic and cholinergic frequencies from total mEPSCs recorded under the different culture conditions. In B27 0.2%-supplemented media, the mEPSCs were due for the largest part to the glutamatergic neurotransmission (glutamatergic: 97.3 ± 3.8%; cholinergic: 3.3 ± 3.8%; *n* = 8) whereas, after chronic treatment of cultures with exogenously-added NGF, a significant decrease in the glutamatergic component of mEPSCs (61.2 ± 1.7%; *n* = 8) was detected in correlation with an increase of the cholinergic one (38.8 ± 1.7%). Values are means of at least four independent cultures and statistically significant differences were calculated by unpaired Student's *t*-test (^***^p < 0.001).

As shown in Figures 4A,B, the mEPSCs recorded in B27 0.2% septum neurons were almost completely due to the glutamatergic neurotransmission (97.3 ± 3.8%; *n* = 8) because the cholinergic one represented only a small component (3.3 ± 3.8%) of the total excitatory activity. On the contrary, under conditions of B27 0.2%+NGF, the glutamatergic component was significantly lower (61.2 ± 1.7%; *n* = 8; *p* << 0.001 unpaired Student's *t*-test) and the cholinergic component inversely higher (38.8 ± 1.7%; *p* << 0.001 unpaired Student's *t*-test) than those measured in B27 0.2% neurons (Figure 4B), in line with our previous biochemical and morphological data (Figures 1, 2) indicating that the cholinergic neurons thrived when cultured in 0.2% B27 medium in the chronic presence of ectopic NGF.

These results indicate that: (i) septohippocampal neurons cultured for 10–12 D.I.V. in the chronic presence of NGF under conditions of 0.2% B27 supplementation are a highly-enriched population of cholinergic neurons expressing phenotype-specific markers (Figures 1, 2) which acquire a mature neuronal morphology along with NGF-dependent neurophysiological functional properties, just resembling the *in vivo* NGF-responsive TrkA-expressing neurons in CNS.

### NGF withdrawal induces an early, selective and reversible deterioration of cholinergic presynaptic terminals, just resembling the “dying-back”-like mechanism(s) of cell degeneration of basal forebrain circuit occurring *In vivo* during AD neuropathology and caused by neurotrophin starvation

Synapses loss and retrograde “dying-back” axonal degeneration—where loss of terminals ends precede somatic cell death—are characteristic features of several neurodegenerative diseases, including AD, appearing at prodromal stages of pathology in correlation with incipient memory dysfunction (Griffin and Watson, 1988; Mandelkow et al., 2003; Roy et al., 2005; Overk and Masliah, 2014). In cognitively healthy elderly individuals with suspected non-Alzheimer's disease pathophysiology (SNAP) or in patients with amnestic MCI, cholinergic basal forebrain neurons may be viable, although dysregulated, but do not display a massive and frank loss making them a valuable target of NGF-based therapeutic approaches aimed to prevent and/or delay the cognitive deterioration starting from the earliest stages of symptomology (Mufson et al., 2008). Therefore, with the intent of investigating the earliest and potentially reversible molecular events that occur within 12 h following withdrawal of NGF and compromise AD neurons prior to initiation of cell death, mature 10–12 D.I.V., septal cholinergic-enriched cultures grown continuously in 0.2% B27 media in the presence of exogenous NGF (100 ng/ml) from plating were deprived of their trophic support for different periods of time (−1.5, −3, −6, −12, −24 h). By Western blotting analysis on total protein extracts, we evaluated the steady-state levels of different markers of synaptic and cytoskeleton compartments which are known to be greatly affected during the initial NGF-dependent “dying-back”-like mechanisms of cell degeneration occurring *in vivo* at the earliest stages of AD neuropathology (Stokin and Goldstein, 2006; Kanaan et al., 2013).

**Figure 5:**
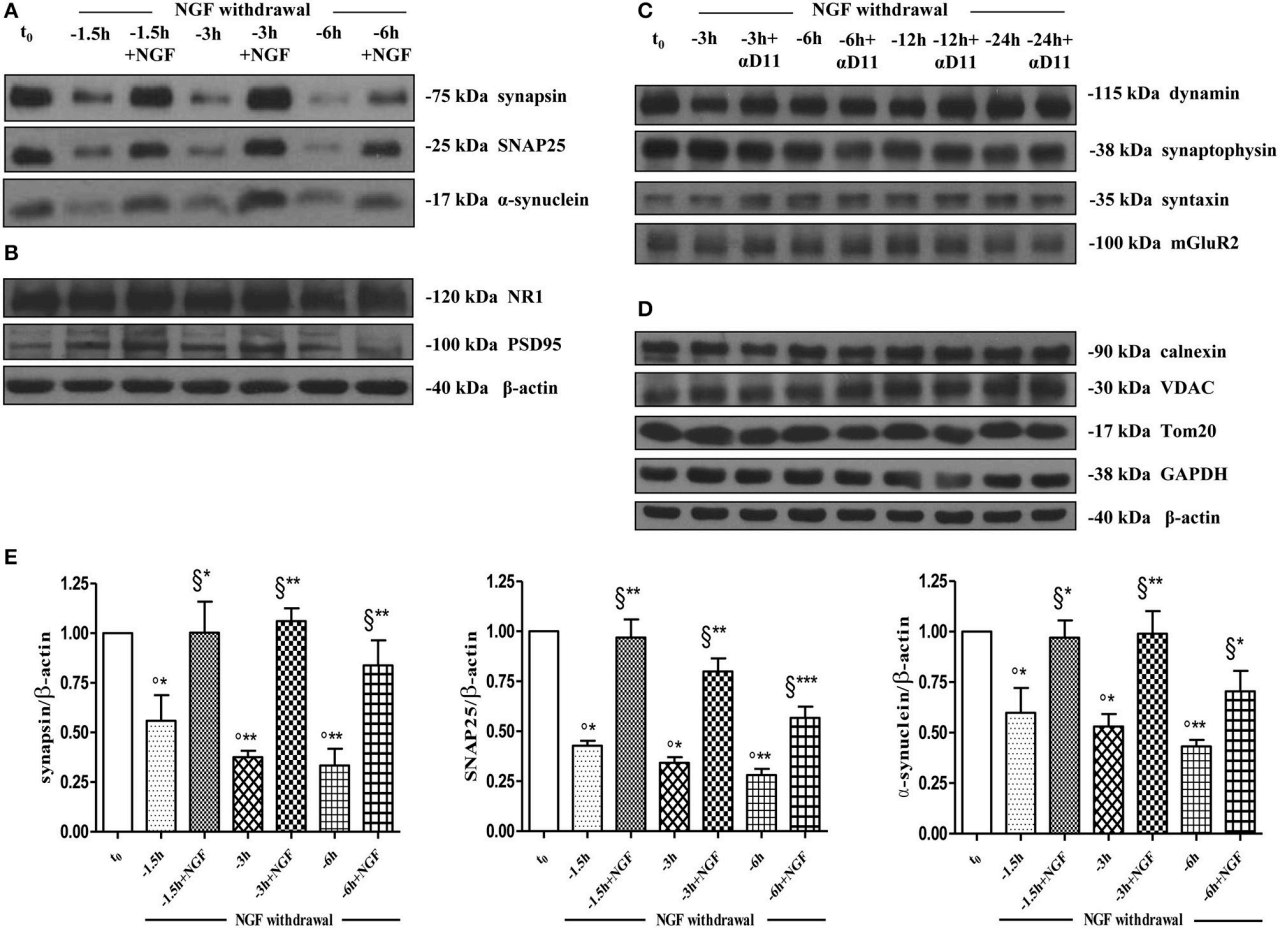
**Early and selective presynaptic deficits of cholinergic presynaptic terminals occur at early time points of NGF withdrawal in septo-hippocampal primary neurons in the absence of global changes in other synaptic and non-synaptic proteins. (A–E)** Septal cholinergic-enriched cultures grown continously in 0.2% B27 media in the presence of exogenous NGF (100 ng/ml) from plating were deprived of their trophic support for different periods of time (−1.5, −3, −6 h) in the presence or in absence of specific neutralizing anti-NGF αD11 monoclonal antibody (800 ng/ml). At the time points indicated, cell were either harvested or re-exposed to NGF (100 ng/ml) and further kept up to 24 h when they were eventually collected. Equal amounts of total protein extract (40–80 μg) were resolved on SDS-PAGE gels and immunoblots were probed with antibodies directed against relevant presynaptic proteins **(A–C)** -including synapsin I, synaptosomal-associated protein 25 (SNAP-25), α-synuclein, dynamin, synaptophysisn, syntaxin, mGluR2-, post-synaptic markers **(B)** -such as N-methyl-D-aspartate glutamate receptor 1 (NR1) and postsynaptic density protein 95 (PSD95)- and not-synaptic proteins located in cytosol, endoplasmic reticulum and mitochondria **(D)** -glyceraldehyde 3-phosphate dehydrogenase (GAPDH), calnexin, voltage-dependent anion channel (VDAC/Porin), translocase of outer membrane (Tom20). Cropped representative WB are shown. Molecular weights are indicated on the right of the blots and expressed in kDa. Densitometric quantification of immunoreactivity levels **(E)** was calculated by normalizing the values on the intensity of β-actin, as loading control for each sample/lane. Values are mean ± SEM of at least nine independent experiments and are expressed with respect to B27 0.2% control neurons at *t* = 0 (t_0_) (°) and at corresponding time (*t* = −1, −3, −6 h) of NGF deprivation (§). Statistically significant differences were calculated by unpaired-two tailed *t*-Student's test (^*^p < 0.05, ^**^p < 0.01, and ^***^p < 0.0001).

**Figure 6:**
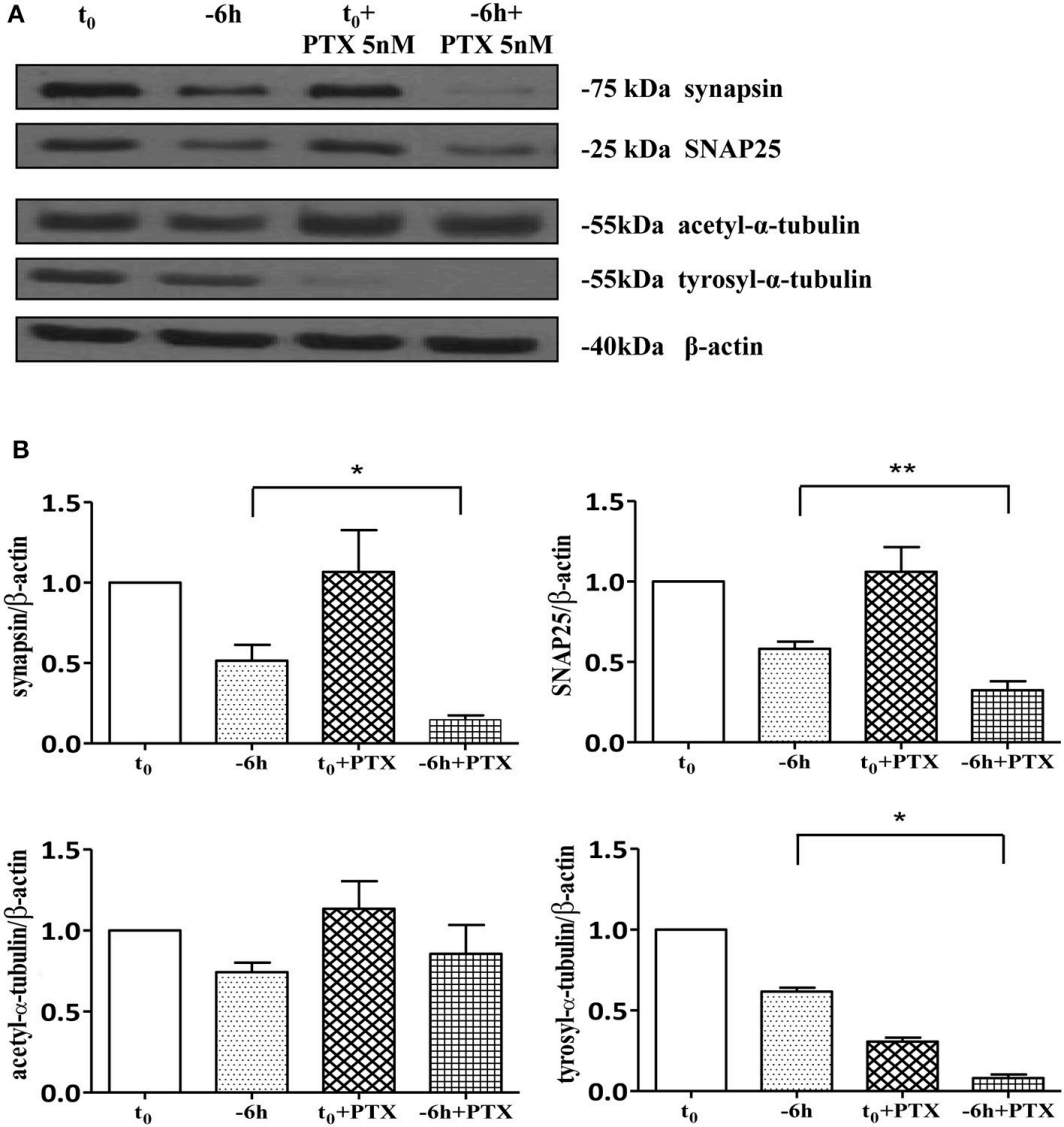
**Loss of presynaptic terminals in NGF-deprived cholinergic neurons is accompanied by pathological alterations in microtubule-dependent axonal stability. (A,B)** Representative blots (*n* = 5) of detergent lysates from septal cholinergic-enriched cultures which were grown continously in 0.2% B27 media in the presence of exogenous NGF (100 ng/ml) from plating and then deprived of their trophic support for 6 h. At time points indicated, cell were either harvested or incubated with 5 nM paclitaxel, a microtubule-stabilizing drug, and further kept up to 24 h when they were eventually collected (F). Densitometric quantification of immunoreactivity levels (G) was calculated by normalizing the immunoreactive signals of each band to corresponding β-actin intensities on the same blots. Modifications of tubulin subunits were contextually evaluated, by probing filters with antibodies directed against acetyl (stable)- and tyrosinylated (instable)- tubulin in order to check the cytoskeleton dynamics. Note that, in contrast to re-application of NGF, treatment with paclitaxel is not able to rescue the immunoreactivity signals of the two selected presynaptic proteins following 6 h neurotrophin starvation. Statistically significant differences were calculated by unpaired-two tailed *t*-Student's test (^*^p < 0.05, ^**^p < 0.01).

As shown (Figures 5A–E) and in line with the evidence that presynaptic proteins are not equally affected in human AD brains (Sze et al., 2000; Honer, 2003; Reddy et al., 2005), the withdrawal of NGF caused in this *in vitro* neuronal paradigm an early (starting from −1 h), gradual and significant decline (^*^p < 0.05, ^**^p < 0.01 vs. *t* = 0) in a few relevant presynaptic proteins -such as synapsin I, synaptosomal-associated protein 25 (SNAP-25) and α-synuclein which are essential for neurosecretion and synaptic plasticity, being involved in local trafficking of synaptic vesicles and/or Ca^2+^-triggered neurotransmitter release. In contrast, other proteins located in the same subcellular compartment -such as dynamin, synaptophysin, syntaxin, mGluR2 -were contextually unmodified up to 24 h and even after incubation with αD11, a specific NGF-neutralizing monoclonal antibody (Cattaneo et al., 1988) which has been previously reported to reduce availability and to antagonize its biological functions *in vivo* (Capsoni et al., 2000; Ruberti et al., 2000; Covaceuszach et al., 2008). These progressive dysfunctional changes were not due to a general, non-specific deterioration in protein amount/quality taking place in the absence of any appreciable cell death up to 48 h, as we previously assessed by MTT assay and Western blotting detection of active cleaved caspase-3 (Supplementary Figure 2). Furthermore, the dramatic changes in the regulation of protein expression and/or stability occurring early following NGF deprivation turned out to be strictly limited to presynaptic terminals because: (i) no substantial and simultaneous alteration in the intracellular amounts of two major postsynaptic proteins—such as N-Methyl-D-aspartate (NMDA) Receptor subunit 1 (NR1) and postsynaptic density protein 95 (PSD95)—was contextually evident when NGF was removed in cholinergic-enriched septal primary neurons; (ii) the expression levels of several not-synaptic proteins—including specific markers of endoplasmic reticulum and mitochondria such as calnexin, VDAC, Tom20 located in different subcellular compartments of cell-body—were also unaffected in these cultures despite prolonged (−24 h) antibody-mediated immunodepletion of NGF trophic support. Only after 48 h of NGF removal and in concomitance with the appearance of marked deterioration signs characterized by an evident neurite degeneration and pre-apoptotic fragmented and heterochromatic nuclei, we detected a significant and general drop in amounts of synaptic markers in this *in vitro* neuronal paradigm (data not shown). Interestingly, we did not measure any change in immunoreactivity of either pre- or post-synaptic proteins when sister basal forebrain primary neurons chronically grown for the same length of time (10–12 D.I.V.) in the presence of NGF but in conditions of rich media (2% B27+NGF experimental group) were deprived of their trophic support up to 24 h (Supplementary Figure 3) in support of the notion that these presynaptic events occurring under condition of 0.2% B27 took place for the large part in the cholinergic-enriched and then NGF-dependent population. In agreement with our previous results (Figures 1, 2), these findings clearly demonstrated that the present newly-developed culture protocol (0.2% B27+NGF for 10–12 D.I.V.) really enriched with a good degree of homogeneity for a consistent, genuine and highly NGF-responsive population representing thus an useful tool for more specific *in vitro* assessment of the early AD-relevant, cholinergic-based processes associate with the loss in NGF/TrkA signaling. In fact, the number of non-cholinergic neurons—which are not sensible to NGF and greatly differ in proportion between cultures grown under the two diverse conditions of B27 supplementation (Figures 1, 2)—was not obviously decreased following neurotrophin starvation, thus taking into account of the significant experimental differences observed. In striking similarity with the *in vivo* observations that is the initial loss in synaptic contacts occurring at the earliest stages of typical AD neuropathology which triggers the following “dying-back” degeneration progressing retrogradely from distal terminal ends to perikaryon and nucleus (Kanaan et al., 2013), this prompt presynaptic failure was not secondary to microtubule network disassembly but preceded the axonal degeneration because: (i) no significant post-translational modifications of tubulin subunits which critically control the cytoskeleton dynamics were concomitantly found *in vitro* up to 6 h of NGF withdrawal, as assessed by Western blotting analysis with antibodies against acetyl (stable)- and tyrosinated (instable)- tubulin (data not shown); (ii) NGF-deprived septal neurons were insensible to pharmacological treatment with known microtubule-stabilizing drugs as evidenced by the observation that the signal intensity of SNAP25, synapsin I and α-synuclein was not rescued when cultures were 30 min preincubated with 5 nM paclitaxel before the NGF withdrawal (Figures 6A,B). Importantly when cholinergic neurons grown in 0.2% B27 media in the chronic presence of exogenous NGF, were deprived of their trophic support for increasing periods of time (−1,−3,−6,−12,−24 h) and then re-exposed to it up to 24 h, we found out that these NGF-dependent presynaptic deficits were virtually abrogated (Figures 5A–E) for the reason that the loss in protein amounts of synapsin I, SNAP25 and α-synuclein was strongly suppressed by its *de novo* external re-application up to 6 h from initial withdrawal (^*^p < 0.05, ^**^p < 0.01 vs. corresponding NGF-deprived sample). However, the control of NGF on the cholinergic pre-synaptic stability was reversible but in a time-dependent manner because the dramatic decline in endogenous levels of these three relevant neurosecretory proteins was no longer sensitive to its exogenous re-application if it was delayed for 12 or 24 h (data not shown), indicating the existence of a critical and restricted period after that most cholinergic neurons become independent on their specific target-derived growth factor and irreversibly committed to degenerate.

Collectively, our results demonstrate that: (i) a marked, simultaneous and sequential downregulation in the expression levels of several distinct presynaptic proteins involved in vesicles trafficking and neurotransmitter release -such as synapsin I, SNAP25 and α-synuclein- occurs in mature cholinergic septal cultures at early time-points following the NGF withdrawal; (ii) these NGF-deprived *in vitro* neurons, just as those which selectively degenerate in the early stages of AD neuropathology owing to alterations in NGF/TrkA survival signaling, retain their ability to respond and re-activate specific biochemical and functional NGF-stimulated cell pathway(s) but only for a critical period of time.

### Pharmachological disruption of TrkA signaling abolishes the NGF-induced effects on presynaptic markers and function

**Figure 7:**
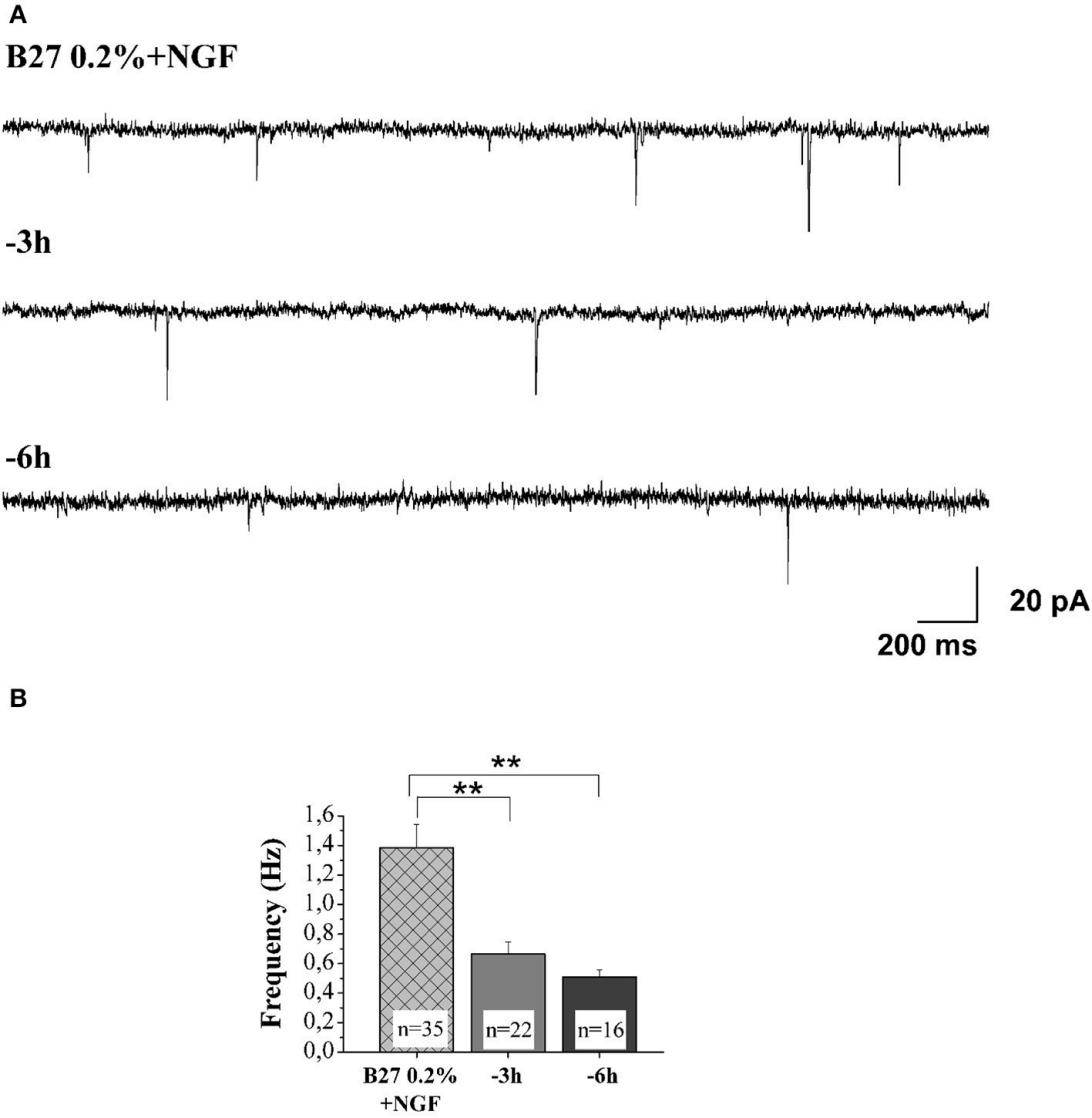
**NGF withdrawal provoked a rapid and time-dependent reduction of excitatory neurotransmitter release in septal-cholinergic primary neurons. (A)** Representative traces of septal cholinegic cultures grown for 10–12 D.I.V. in B27 0.2%+NGF and deprived for 3 and 6 h of NGF trophic support. **(B)** Bar plots reporting the mean ± SEM frequency of the recorded mEPSCs displayed a significant reduction in mEPSCs frequency detected in *in vitro* cholinergic-enriched neurons following 3 h (0.66 ± 0.08 Hz; *n* = 22) and 6 h of NGF removal (0.5 ± 0.05 Hz; *n* = 16) in comparison with B27 0.2%+NGF control ones (1.38 ± 0.16 Hz; *n* = 35). Values are means of at least four independent cultures and statistically significant differences were calculated by One-Way ANOVA followed by Bonferroni's correction (^**^p < 0.01 vs. 0.2% B27+NGF).

An imbalance between the TrkA-mediated survival signaling and the p75NTR-mediated pro-apoptotic signaling has been hypothesized to compromise the cholinergic basal forebrain neuronal function(s) during the prodromal stages of AD (Counts and Mufson, 2005; Mesulam, 2013). Consistently, a partial activation of TrkA can upregulate the cholinergic phenotype of the basocortical projection system and improve cognition, in the absence of p75NTR activation (Bruno et al., 2004). Next, in order to study the pathological relevance of these NGF-dependent alterations in the context of *in vivo* AD neurodegeneration and considering that the MS/DB cholinergic neurons—but not noncholinergic ones—largely (>95%) express the high-affinity TrkA receptor, which makes them selectively sensitive to direct action of NGF (Sobreviela et al., 1994; Sanchez-Ortiz et al., 2012), we investigated whether the potent actions of NGF in controlling both the steady-state levels of selected presynaptic proteins and the neurotransmission were causally linked and occurred via activation of signaling of TrkA whose expression levels we detected to be significantly up-regulated under 0.2% B27+ NGF in *in vitro* conditions (Figures 1A,B). First of all, having established that the withdrawal of NGF in 0.2% B27 cholinergic neurons was able to cause a rapid and time-dependent downregulation in the expression levels of several presynaptic proteins involved in vesicles trafficking, we set about checking whether the NGF-dependent increase in the frequency of mEPSCs—which is associated to the augmented probability of exocytosis of synaptic vesicles at presynaptic terminals rather than to postsynaptic responses (Huh et al., 2008)—was also concomitantly affected in this *in vitro* paradigm upon neurotrophin starvation. As shown in Figures 7A,B and in correlation with the rapid and selective decline in immunoreactivity of SNAP25, synapsin I and α-synuclein as we detected by Western blotting analysis (Figures 5A–E), we uncovered that- in the absence of any change in cell viability which occurred only after 48 h of deprivation (Supplementary Figure 1)- the averaged frequency of mEPSCs was significantly lower in 0.2% B27 cholinergic neurons following short-term removal of exogenous NGF for 3–6 h (3 h: 0.66 ± 0.08 Hz, *n* = 22; 6 h: 0.59 ± 0.05 Hz, *n* = 16; *p* < 0.001, One-Way ANOVA) in comparison with control B27 0.2%+NGF cultures (1.39 ± 0.16 Hz, *n* = 35). On the contrary, the mean amplitude as well as the mean area and the kinetic parameters of the mEPSCs were not modified by NGF deprivation (data not shown), in line with previous findings (Huh et al., 2008).

**Figure 8:**
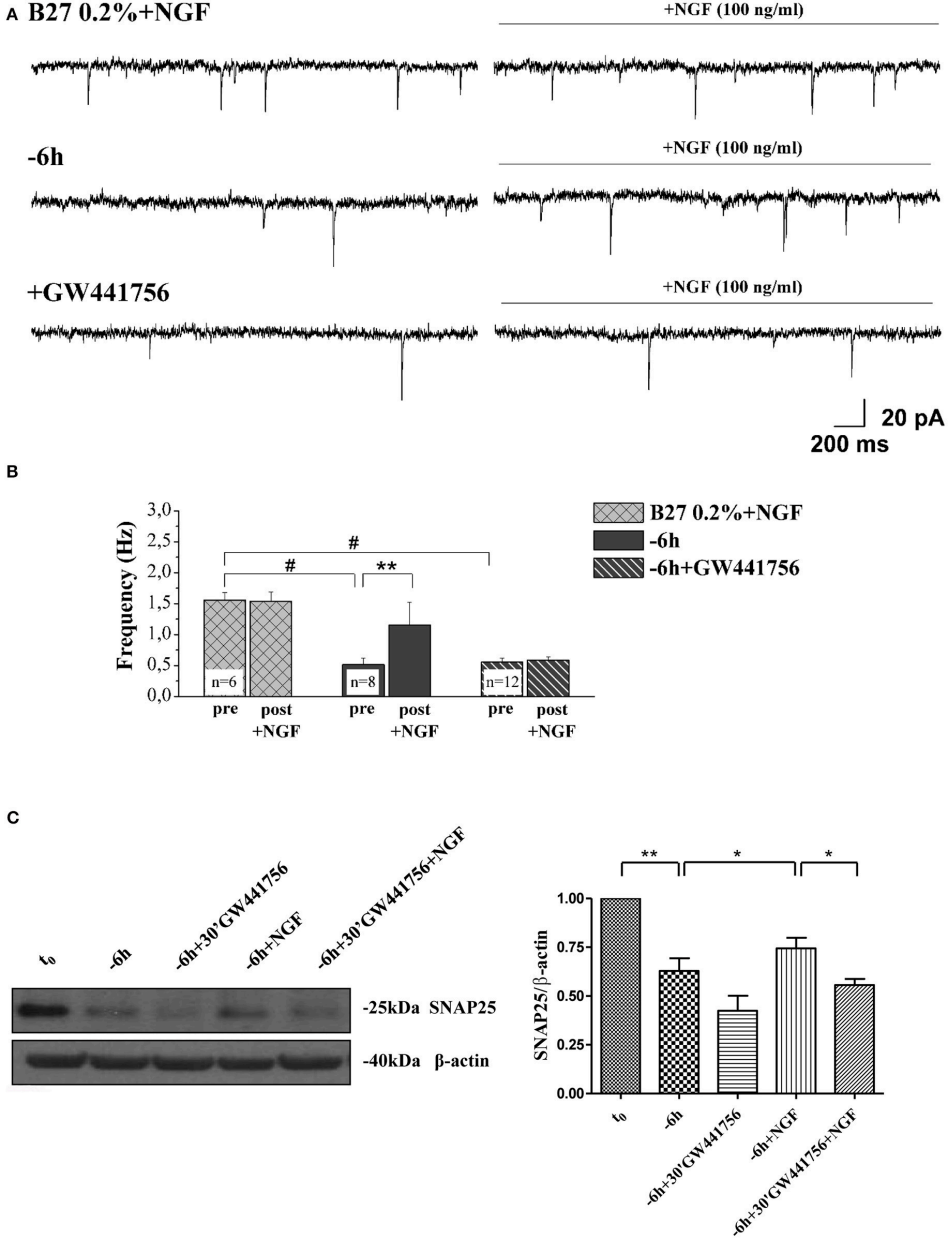
**NGF induced a facilitation of presynaptic excitatory neurotransmitter release from cholinergic septal neurons by acting on TrkA receptor. (A)** Representative traces of septal neurons grown for 10–12 D.I.V. in B27 0.2%+NGF, deprived for 6 h of NGF, in the presence and in the absence of GW441756 TrkA inhibitor (15 μM), in basal settings (left) and following 3 min of NGF acute perfusion (100 ng/ml; right). **(B)** Bar plots report the mean ± SEM frequency of the mEPSCs recorded under different experimental conditions, pre and post NGF perfusion. NGF acute perfusion significantly increased the mEPSC frequency in 6 h deprived neurons (pre: 0.51 ± 0.1 Hz; post: 1.16 ± 0.36 Hz, *n* = 8; *p* < 0.01, paired Student's *t*-test) but was not effective in B27 0.2%+NGF neurons (pre: 1.55 ± 0.11 Hz; post: 1.54 ± 0.15 Hz, *n* = 6; *p* > 0.05 paired Student's *t*-test) and when GW441756 TrkA inhibitor was added to solutions (pre: 0.55 ± 0.07 Hz; post: 0.58 ± 0.05 Hz, *n* = 12; *p* > 0.05, paired Student's *t*-test). Values are means of at least three independent cultures and statistically significant differences were calculated by paired two-tailed Student's *t*-test (^**^p < 0.01, pre- vs. post-NGF perfusion in 6 h-deprived neurons) and by One-Way ANOVA followed by Bonferroni's correction (hash (#) *p* < 0.001 vs. B27 2%+NGF). **(C)** Representative blots (*n* = 3) of total lysates from septal cholinergic-enriched cultures which were grown continously in 0.2% B27 media in the presence of exogenous NGF (100 ng/ml) from plating and then deprived of their trophic support for 6 h. At time points indicated, cell were either harvested or re-exposed to NGF (100 ng/ml) in the presence or absence of GW441756 TrkA inhibitor (15 μM) up to 24 h when they were eventually collected. Densitometric quantification of immunoreactivity levels of presynaptic SNAP25 was calculated by normalizing the immunoreactive signals of each band to corresponding β-actin intensities on the same blots. Statistically differences were calculated by paired two-tailed *t*-Student's test (^*^p < 0.05; ^**^p < 0.01).

Next, in order to assess the potential causal relationship between these two concomitant NGF-triggered events, 0.2% B27+NGF cultures deprived for 6 h of their trophic support were re-exposed to it- in the presence or absence of GW441756 a potent, selective and cell-permeable inhibitor of TrkA (Wood et al., 2004)- and then the expression levels of SNAP25, synapsin I and α-synuclein along with the efficacy in neurotransmitter release were assessed in parallel, by Western blotting analysis and electrophysiological recording of mEPSCs respectively (Figure 8). Figure 8A displays the representative mEPSCs recorded at the holding potential −60 mV in B27 0.2%+NGF neurons following 6 h deprivation before and after the acute perfusion of exogenous NGF (100 ng/ml), in the presence or in absence of GW441756 TrkA inhibitor (15 μM, −20 min). As shown in Figure 8B, the averaged mEPSCs frequency measured in cholinergic neurons cultured in B27 0.2% supplementation in the chronic presence of NGF following 6 h deprivation was significantly enhanced by its *de novo* acute perfusion (pre: 0.51 ± 0.1 Hz; post: 1.16 ± 0.36 Hz, *n* = 8; *p* < 0.01, paired Student's *t*-test) whereas not significant difference was detected in comparison to B27 0.2%+NGF controls (*p* > 0.05 unpaired Student's *t*-test) which, as expected, were not sensible to NGF acute perfusion (pre: 1.55 ± 0.11 Hz; post: 1.54 ± 0.15 Hz, *n* = 6; *p* > 0.05 paired Student's *t*-test). Interestingly the acute effect of NGF infusion on mEPSC frequencies was completely blocked by 20 min pretreatment with GW441756 inhibitor, indicating that NGF exerted its presynaptic facilitation of neurotransmitter release via TrkA-mediated mechanism(s) (pre: 0.55 ± 0.07 Hz; post: 0.58 ± 0.05 Hz, *n* = 12; *p* > 0.05, paired Student's *t*-test). Furthermore and more importantly we found out that the concomitant up-regulation in expression levels of presynaptic marker SNAP25 evoked by re-application of exogenous NGF to 6 h-deprived cholinergic neurons was also greatly prevented by blocking the TrkA/NGF signaling with GW441756 inhibitor, as shown by Western blotting on total protein extracts from treated cultures (Figure 8C). Similar results were detected for the other two presynaptic proteins, synapsin I and α-synuclein (data not shown). The efficacy of GW441756 in negatively affecting the initial event of NGF/TrkA signal transduction was also checked by probing with specific antibody against TrkA (Y490) phosphorylation (data not shown) which is largely known to occur rapidly upon exposure to NGF in primary septal neurons (Triaca et al., 2016). These results clearly demonstrated that the combined modulatory actions of NGF on the presynaptic morphology and neurosecretory function(s) of *in vitro* cholinergic septal primary neurons were directly and causally linked via the stimulation of NGF/TrkA signaling (Figures 8A–C), pointing out the pathological relevance of the lack in NGF availability in the earliest synaptic deficits occurring at the onset of AD progression.

Taken together, our biochemical and electrophysiological results demonstrate that blocking NGF/TrkA signaling decreases the probability of exocytosis of synaptic vesicles at presynaptic terminals likely by downregulating the steady-state levels of relevant presynaptic proteins which critically control the vesicular trafficking and neurotransmission, including synapsin I, SNAP25 and α-synuclein.

## Discussion

A growing number of cellular and animal studies suggests that AD is a synaptic failure and begins with subtle alterations of hippocampal synaptic efficacy prior to frank neuronal degeneration (Selkoe, 2002). Compelling *in vitro* and *in vivo* evidence supports a pivotal role for an imbalance between TrkA-mediated survival signaling and p75NTR-dependent pro-apoptotic pathway in triggering the selective atrophy of cholinergic septo-hippocampal population occurring in prodromal stages of AD neuropathology (Counts and Mufson, 2005; Niewiadomska et al., 2011; Schliebs and Arendt, 2011; Grothe et al., 2014). Furthermore, morphological investigations carried out on experimentally lesioned animals and on postmortem human brain tissues have demonstrated that most basal forebrain cholinergic neurons, although downregulated in their phenotypic markers, persist in an atrophic state during the AD progression rather than degenerate, being thus suitable to effective drug treatment or neuroprotective strategies aimed to prevent/attenuate the cognitive dysfunction associated to degeneration of their projections to cortical areas (Mufson et al., 2008; Potter et al., 2011). In line with previous reports (Sassin et al., 2000; Mesulam et al., 2004; Grothe et al., 2013; Kilimann et al., 2014), longitudinal studies of anatomical magnetic resonance imaging (MRI) performed on AD patients at different stages of neuropathology have recently confirmed that presymptomatic lesions of cholinergic neurons of nucleus basalis of Meynert precede the earliest memory impairments, indicating that therapeutic strategies targeting the basal forebrain will actually offer promising opportunities in delaying and/or prevent the transition from MCI to frank AD (Potter et al., 2011; Schmitz et al., 2016). In AD brains, the intracerebral level of NGF appears to be stable or even increased in cortex, whereas a significant reduction is detected in basal-forebrain cholinergic population (Mufson et al., 1995; Scott et al., 1995), in support of the conclusion that the early perturbation in NGF/TrkA signaling occurring within cholinergic vulnerable areas is more likely to be due to defective retrograde transport of this target-derived neurotrophin to its responsive neurons (Salehi et al., 2004; Counts and Mufson, 2005). Consistently, infusion of NGF can prevent septal cholinergic neuron death following septo-hippocampal axotomy (Hefti, 1986; Williams et al., 1986; Tuszynski and Gage, 1995) and AD11 transgenic mice engineered to express anti-NGF neutralizing antibody in adulthood display a severe and age-dependent loss of ChAT-positive basal forebrain neurons (Capsoni et al., 2000; Ruberti et al., 2000). Perturbation in the excitatory-inhibitory balance of neural circuitries early occurs within the adult AD11 hippocampi (Lagostena et al., 2010), just resembling the initial compensatory alteration in neurotransmission observed in AD pathology which progressively contributes itself to exacerbate the correlated cognitive deficits by disrupting of the overall network activity (Palop et al., 2007). In addition to cholinergic loss and hippocampal-dependent memory deficits, the activation of amyloidogenic APP cascade with intra/extracellular accumulation of Aβ peptide(s) along with the dysmetabolism (hyperphosphorylation/cleavage) of tau are also detected in animal and cellular neuronal models undergoing NGF withdrawal which recapitulate a comprehensive AD-like neuropathology (Capsoni et al., 2000; Ruberti et al., 2000; Matrone et al., 2008a,b; Houeland et al., 2010; Amadoro et al., 2011; Triaca et al., 2016). These important findings support the “neurotrophic model” which states that the reduced availability of NGF is more likely to be an upstream driver for *in vivo* AD neurodegeneration, by linking all its typical histopathological signs into a common neurodegenerative cascade (Cattaneo and Calissano, 2012). Furthermore, provided that the potent stimulatory effect of synaptic vesicles exocytosis evoked by NGF on presynaptic nerve endings is unique to cholinergic neurons (Huh et al., 2008), the alterations in NGF/TrkA signaling in this vulnerable septo-hippocampal population may actually play a critical role in promoting the synaptic derangements and neurotransmission deficits occurring in MCI and at early stage of AD pathology (Cattaneo and Calissano, 2012). In this framework, any data strengthening the causal molecular link between NGF dyshomeostasis and early-stage AD features could be helpful in assisting the development of more effective neuroprotective and disease-modifying non-invasive cholinergic therapy for the amelioration of AD symptomology.

The present study explores the early synaptic mechanisms underlying the failure in neurotransmission provoked by the lack of NGF trophic supply, which is causally involved in selective degeneration of septo-hippocampal circuit and AD-associated cognitive decline, with the following five pertinent findings. First, we devise a newly-developed culture protocol allowing with a high-degree of homogeneity a selective enrichment in the NGF-responsive cholinergic population at the expense of other noncholinergic septal neurons. By turning to this *in vitro* neuronal paradigm, we show that neurotrophin starvation leads to selective and rapid (−1 h) downregulation in the steady-state levels of three relevant regulators of presynaptic vesicles dynamics located at nerve endings, such as synapsin I, SNAP25 and α-synuclein. Second, we demonstrate that these seminal presynaptic alterations affecting the NGF-dependent cholinergic neurons of septum anticipate the “dying-back” degeneration occurring at prodromal/early stages of AD neuropathology because they are not accompanied by any significant and concomitant change in microtubule-dependent axonal stability and neuronal viability. Third, we reveal that the specific changes in proteome involving the secretory machinery are causally linked to a parallel decline in the NGF-stimulated frequency of spontaneous miniature events, suggesting that cholinergic neurons are functionally downregulated in the absence of NGF being unable to respond to its stimulatory action at the presynaptic level. Fourth, we display that the activation of NGF/TrkA signaling in cholinergic cultures mediates the regulation of the expression levels of selected presynaptic proteins -such as synapsin I, SNAP25 and α-synuclein- and, in turn, of the increase in synaptic neurotransmission. Fifth, we delineate the existence of critical time window after that point most of cholinergic neurons are irreversibly committed to degenerate because the biochemical and functional events above-described are reversible but only within 6 h from removal of NGF. Each step will be further discussed in more detail.

Concerning the newly-developed cell culture method we established (Figures 1–4) it's noteworthy that, although rat (E17-E18) septohippocampal primary cultures—which are routinely kept under 2% B27% or similar growth conditions—were previously used in biochemical, immunocytochemical and functional *in vitro* studies (Hartikka and Hefti, 1988a,b; Alderson et al., 1990; Svendsen et al., 1994), detailed molecular analyses have documented that after 10–12 D.I.V. only 1.7–2.1% of all MAP-2 positive cells in *in vitro* basal forebrain neurons are truly cholinergic and NGF-responsive (Hartikka and Hefti, 1988a,b; Oosawa et al., 1999; Auld et al., 2001). Therefore, considering that the very low (less than 5%) percentage of yield of cholinergic neurons following the above-mentioned culture protocol puts important practical limitations which restrain its wide utilization in several fields of applications at single cell-type level, the setting up of optimization methods for reproducing a sufficient amount of homogeneous phenotype-specific population in basal forebrain primary cultures represents an important breakthrough in the reasearch of AD neuropathology. Here, based on immunocytochemical along with Western blotting and electrophysiological data, we report that a reduction in B27 supplementation (0.2%) in the growth media in the chronic presence of exogenously-applied NGF (100 ng/ml) produces severalfold (+36.36%) enrichment in ChAT/TrkA-positive and NGF-dependent cholinergic neurons in 10–12 D.I.V. septal cultures from basal forebrain at the expense of all other non-cholinergic—mainly GABAergic (−38.45%) and glutamatergic (−56.25%)—populations. Relevantly, the more consistent proportion of *in vitro* septo-hippocampal cholinergic neurons obtained following this culture protocol is fully mature, at both morphological and electrophysiological levels, and therefore, turns out to be particularly useful to: (i) investigate more accurately and more specifically the physiopathological cholinergic events occurring at pre-symptomatic stages of AD neurodegeneration with particular regard to the underlying synaptic deficits due to alteration in NGF/TrkA signaling; (ii) test the therapeutic efficacy of external supplementation of NGF trophic support restricted only to degenerating vulnerable basal forebrain ChAT-positive neurons which strictly represent *in vivo* its target population. In line with previous paper (Hartikka and Hefti, 1988b), the contribution of endogenous levels of neurotrophin(s) in this *in vitro* pure neuronal model of NGF deprivation is quite negligible, as demonstrated by the fact that less than 3% of the cell population appears to be of glial nature (astrocytes) after immunocytochemical staining of Glial fibrillary acid protein (GFAP) at 12 days after plating. Furthermore, in support of the finding that the generation of a more sizable NGF-responsive population will allow a more accurate *in vitro* assessment of the AD-related neurodegenerative processes, our preliminary results (Supplementary Figure 4) demonstrate that the APP metabolism is greatly influenced following NGF withdrawal in this novel neuronal paradigm. In line with previous findings reporting a modulatory effect of NGF signaling on APP expression (Araki and Wurtman, 1998; Matrone et al., 2008b), a significant upregulation in expression of its three isoforms of ~110, ~120, and ~130 kDa was detected in 0.2% B27+NGF septo-hippocampal neurons upon 48 h of neurotrophin starvation, as shown by Western blotting analysis with 22C11 antibody directed against the N-terminal epitope (aa 66–81) of holoprotein. Furthermore, by probing with 4G8 antibody which recognizes the residues 18–23 of the Aβ sequence, we revealed that the NGF-triggered APP amyloidogenic processing is activated in cholinergic-enriched 0.2% B27+NGF septohippocampal neurons following 48 h of withdrawal whereas the immunoreactivity level of the diagnostic carboxyl-terminal βCTF fragment of 14 kDa appears to be below the threshold of detection in 2% sister coltures processed at the same point-time and in the same experimental manner. In line with *in vivo* evidence concerning the potential initial role of basal forebrain degeneration in AD onset/progression (Beach et al., 2000; Roher et al., 2000; Grothe et al., 2013; Schmitz et al., 2016), these results help to clarify the important issue concerning the causal role of the loss in NGF-stimulated TrkA-mediated signaling in the activation of the pathogenetic amyloid cascade, suggesting that NGF deprivation is actually able to directly and locally trigger the amyloidogenic APP processing in responsive cholinergic-enriched basal forebrain neurons. In view of these investigation and in agreement with our previous papers (Cattaneo and Calissano, 2012; Triaca et al., 2016), it is tempting to speculate that the early degeneration of cholinergic basal forebrain neurons can initiate the Aβ pathology which, in turn, *in vivo* trans-synaptically spread to the neocortex. Alternatively, as suggested by other investigators (Boncristiano et al., 2002; Salehi et al., 2004; Schliebs and Arendt, 2006), the production of independently-generated Aβ peptide may influence the integrity of TrkA-bearing neurons resulting into disruption of NGF signaling and, then, of maintenaince of cholinergic basal forebrain neurons.

By taking advantage of this newly-developed *in vitro* culture protocol, our biochemical and electrophysiological approaches of Western blotting analyses and spontaneous excitatory postsynaptic currents (EPSCs) recordings (Figures 5–8) on cholinergic-enriched septal neurons clearly demonstrate that the reduction of NGF bioavailability is early paralleled by a selective down-regulation in expression levels of three relevant presynaptic proteins -such as synapsin I, SNAP25 and α-synuclein- which limits the responsiveness of presynaptic terminals to NGF-stimulated synaptic vesicles exocytosis. The morphological and functional control of NGF on presynaptic terminals occurs via TrkA-mediated mechanisms and its deregulation is reversible within a narrow temporal window because NGF-deprived cholinergic neurons remain able to respond to delayed NGF somministration until 6 h after its withdrawal. In agreement with these observations, it has been reported that the ability of NGF in reverting the neurodegenerative phenotype in AD11 mice following its *in vivo* chronic delivery through olfactory bulb is less effective after 6 months of age (Capsoni et al., 2002) and that the retrograde NGF/TrkA signaling is necessary and sufficient to promote the synapses assembly in postganglionic sympathetic neurons (Sharma et al., 2010). Besides, the work we have presented is also consistent with compelling evidence showing that the changes in synaptic structure and function are early markers of age-linked AD (Terry et al., 1991; Selkoe, 2002; Scheff et al., 2006, 2007; Nimmrich and Ebert, 2009) and that the levels of NGF are significantly decreased in the cholinergic basal forebrain of AD brains (Mufson et al., 1995). NGF deficits are proved to be correlated both to amyloidogenic APP processing (Capsoni et al., 2002; Matrone et al., 2009) and to synaptic impairment (Lagostena et al., 2010) in cellular and animal AD models, further supporting the “neurotrophic unbalance” hypothesis as underlying trigger of AD-like neurodegeneration (Francis et al., 1999; Cattaneo and Calissano, 2012). Interestingly, in addition to loss of synapses, synaptic function is also affected in AD pathology by decrements in transcript species related to presynaptic vesicles trafficking, as shown by gene expression profiling on affected brain areas (Mufson et al., 2002; Coleman and Yao, 2003; Yao, 2004; Ginsberg et al., 2006). The patterns (amplitude/frequency) of spontaneous mEPSC, playing important roles in maintaining functional connections of synapses, has been suggested to tag selected synapses for the occurrence of metaplasticity, reflecting long-term consequences of prior synaptic activity or experience (Zhang et al., 2005). Furthermore, our findings on the coordinate NGF-dependent regulation of selected presynaptic proteins involved in vesicles trafficking and of neurontransmitter exocytosis are supported by previous studies reporting that the application of either NGF or TrkA NGF-mimetic agonists successfully rescues the loss of cortical cholinergic boutons in impaired old rats (Cuello et al., 2007), in AD mice models (Aboulkassim et al., 2011; Capsoni et al., 2012) and in humans (Tuszynski et al., 2005; Rafii et al., 2014). Interestingly, the CSF increased levels of several synaptic proteins -including those involved in vesicles trafficking and neurotransmitter exocytosis- have recently turned out to be useful in monitoring the early synaptic degeneration in correlation with the rate of cognitive impairment of AD subjects at pre-symptomatic stages of neuropathology, representing thus a novel potential diagnostic and prognostic biomarker in clinical practice (Davidsson et al., 1999; Jahn et al., 2011; Kvartsberg et al., 2015; Öhrfelt et al., 2016).

Taken together, our studies demonstrating that NGF deprivation acts directly and locally on neurosecretory function of cholinergic presynaptic terminals via TrkA-activation by controlling the steady state levels of synapsin I, SNAP25 and α-synuclein provide for the first time an *in vitro* system model to elucidate the direct interplay between the lack of trophic supply and the early synapses derangement which is causally associated with neurodegeneration and aging. Furthermore, our findings can hopefully have important clinical implications in the fields of therapeutical *in vivo* NGF delivery in humans because not only constitute a molecular rationale for the existence of its limited therapeutic time window but also offer a prime useful presynaptic-based target with the intent of extending its neuroprotective action in AD intervention.

## Ethics statement

This study was performed in accordance with the guidelines established by the European Communities Council (Directive 2010/63/EU of 22 September 2010) and accepted by the Italian Ministry of Health and approved by the Ethical Committee on animal experiments of EBRI “Rita Levi-Montalcini” Foundation (Rome, Italy).

## Author contributions

VL performed morphological, biochemical experiments; SC performed electrophysiological recordings; MC prepared primary neuronal cultures; CZ contributed analytic tools and helped through discussion; GA designed the research, supervised the experiments and wrote the manuscript; PC designed the research and contributed through discussion.

## Funding

This research was supported by PRIN 2010–2011 (prot. 2010M2JARJ-003) to GA and Magnetic Diagnostic Assay for neurodegenerative diseases H2020-ICT-2016-2017 “SSI - Smart System Integration” Proposal 732678 SEP 210349930.

### Conflict of interest statement

The authors declare that the research was conducted in the absence of any commercial or financial relationships that could be construed as a potential conflict of interest.
