# Supplementary figures and images for: Impaired NGF/TrkA Signaling Causes Early AD-Linked Presynaptic Dysfunction in Cholinergic Primary Neurons
Source: Front Cell Neurosci. 2017 Mar 15;11:68. doi: 10.3389/fncel.2017.00068 (PMC5350152; doi:10.3389/fncel.2017.00068)

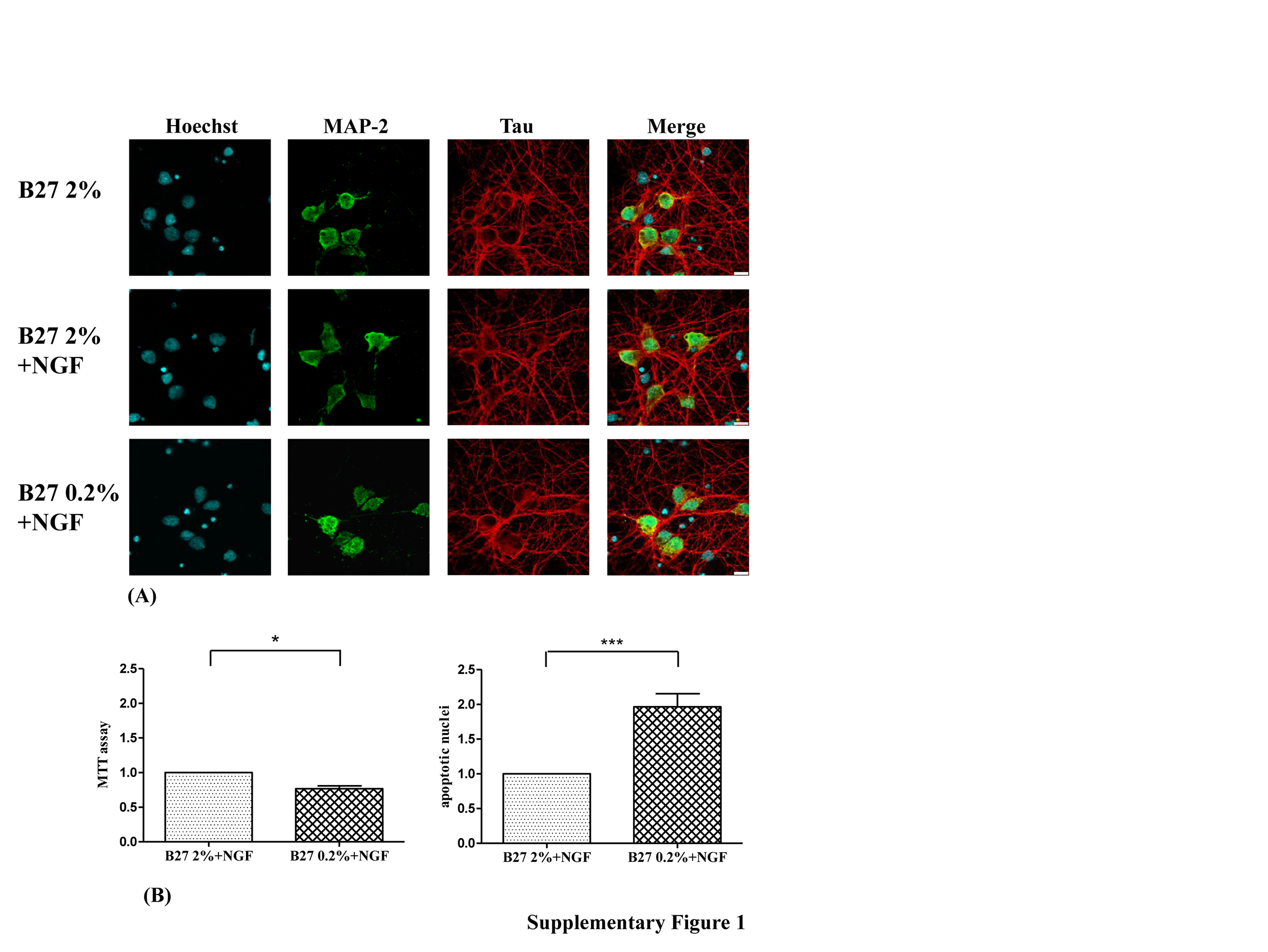

Supplement: Supplementary file 1 [file Image1.tif]

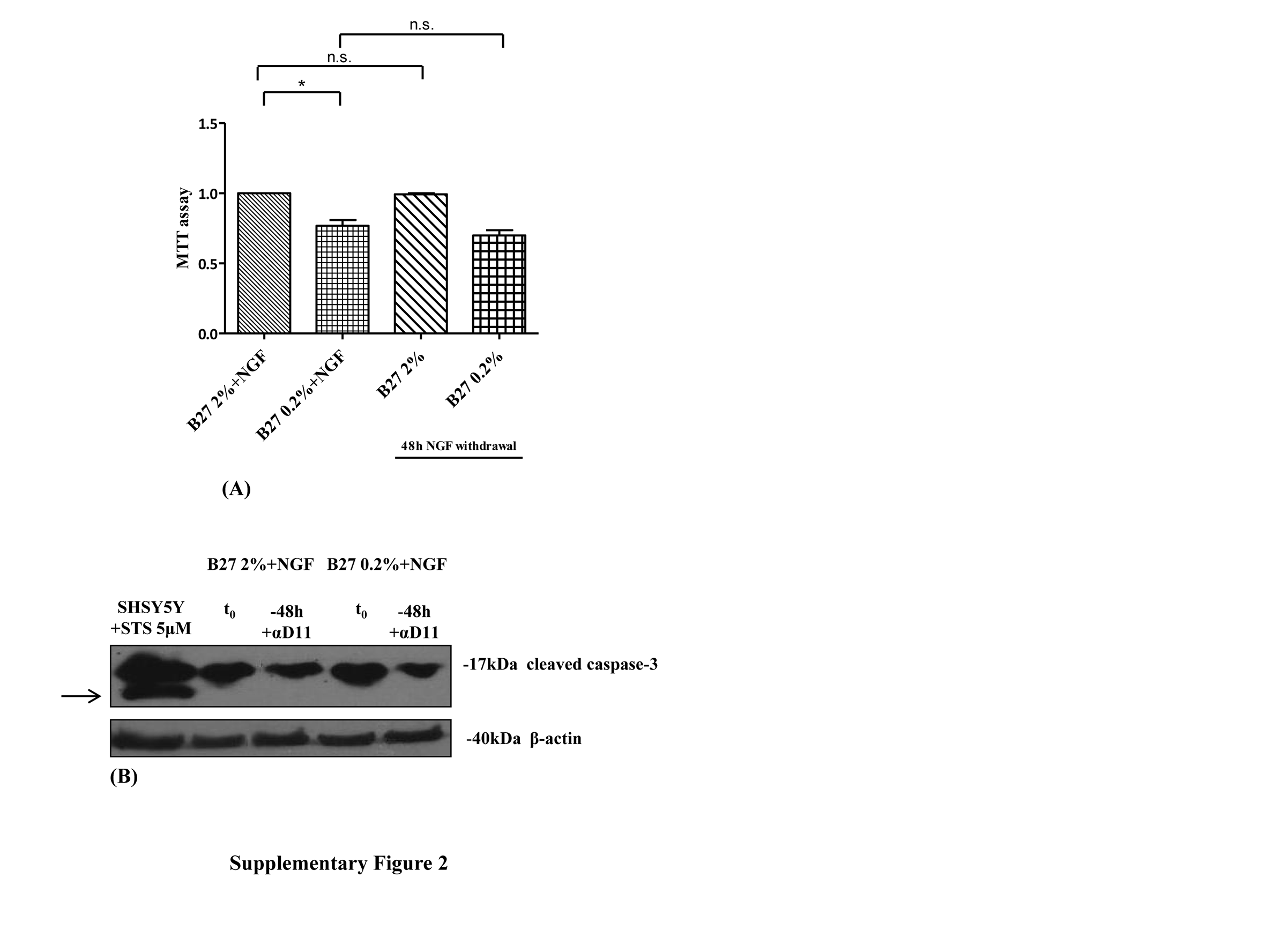

Supplement: Supplementary file 2 [file Image2.tif]

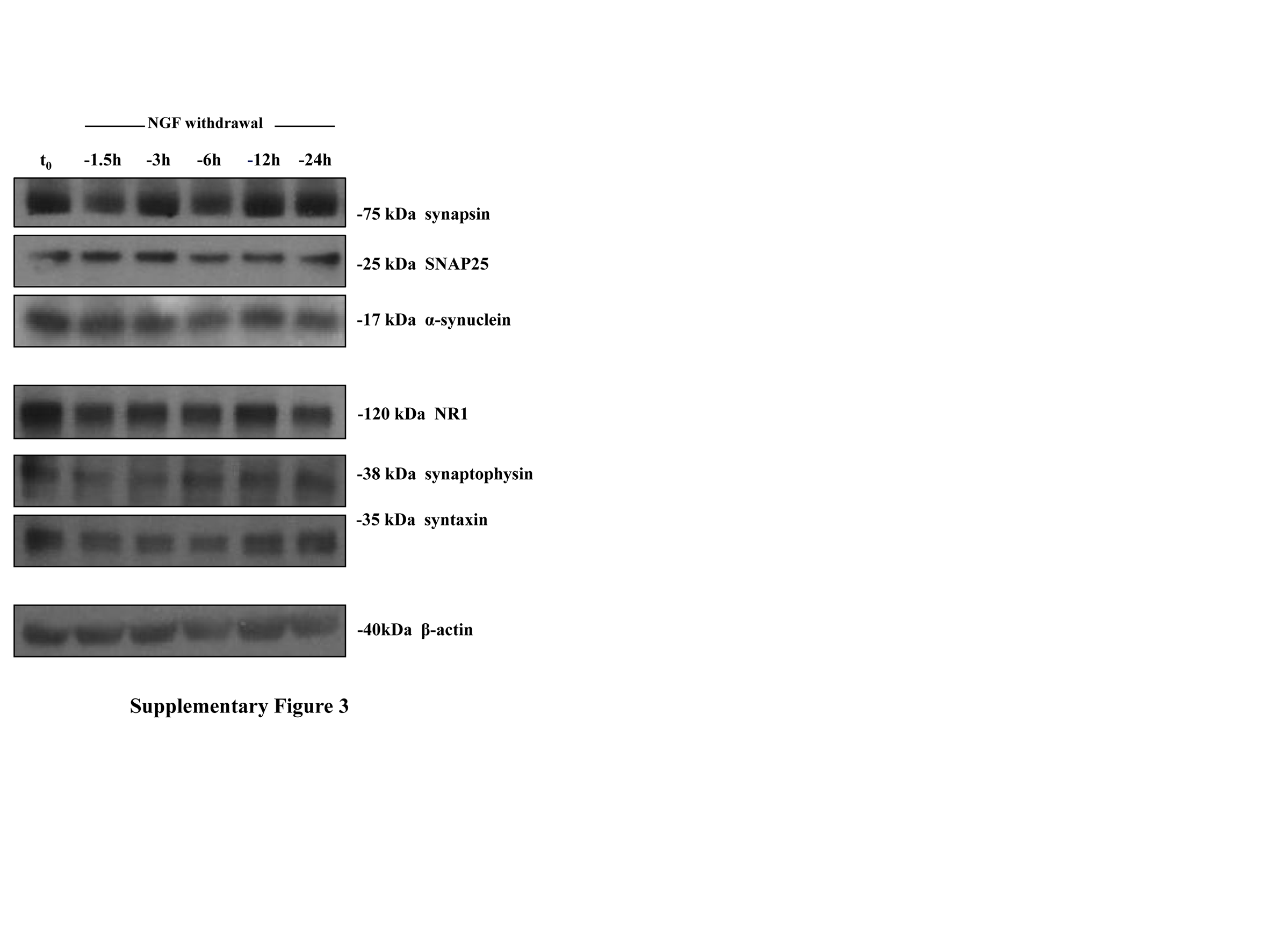

Supplement: Supplementary file 3 [file Image3.tif]

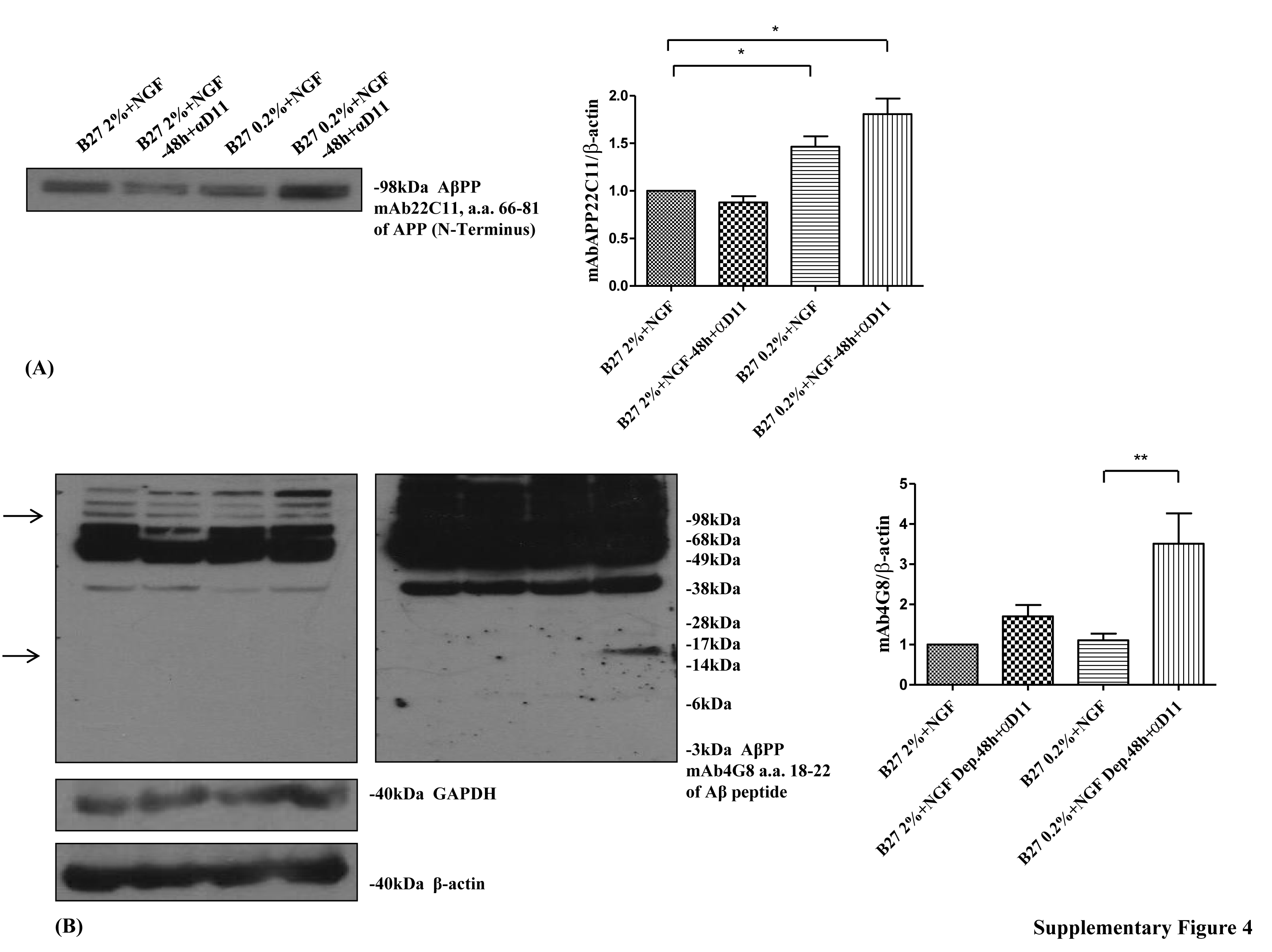

Supplement: Supplementary file 4 [file Image4.tif]
